# Epigenetic regulation of histone modifications in glioblastoma: recent advances and therapeutic insights

**DOI:** 10.1186/s40364-025-00788-w

**Published:** 2025-05-31

**Authors:** Li Zhang, Yang Yang, Yanchu Li, Chenyu Wang, Chenbin Bian, Hongbin Wang, Feng Wang

**Affiliations:** 1 Division of Head & Neck Tumor Multimodality Treatment, Cancer Center, West China Hospital, Sichuan University, Chengdu, Sichuan, China; 2 Division of Abdominal Tumor Multimodality Treatment, Cancer Center, West China Hospital, Sichuan University, Chengdu, Sichuan, China; 3 Yuexiu District, First Affiliated Hospital of Sun Yat-Sen University, Zhongshan 2 Road, Guangzhou City, Guangdong Province, China

**Keywords:** Glioblastoma, Histone modifications, Histone acetylation, Histone methylation, Glioblastoma chemoresistance

## Abstract

Glioblastoma (GBM) is the most common primary malignant brain tumor, characterized by its aggressive behavior, limited treatment options, and poor prognosis. Despite advances in surgery, radiotherapy, and chemotherapy, the median survival of GBM patients remains disappointingly short. Recent studies have underscored the critical role of histone modifications in GBM malignant progression and therapy resistance. Histones, protein components of chromatin, undergo various modifications, including acetylation and methylation. These modifications significantly affect gene expression, thereby promoting tumorigenesis and resistance to therapy. Targeting histone modifications has emerged as a promising therapeutic approach. Numerous pre-clinical studies have evaluated histone modification agents in GBM, including histone deacetylase inhibitors and histone methyltransferase inhibitors. These studies demonstrate that modulating histone modifications can alter gene expression patterns, inhibit tumor growth, induce apoptosis, and sensitize tumor cells to conventional treatments. Some agents have advanced to clinical trials, aiming to translate preclinical efficacy into clinical benefit. However, clinical outcomes remain suboptimal, as many agents fail to significantly improve GBM patient prognosis. These challenges are attributed to the complexity of histone modification networks and the adaptive responses of the tumor microenvironment. This review provides a comprehensive overview of epigenetic regulation mechanisms involving histone modifications in GBM, covering their roles in tumor development, tumor microenvironment remodeling, and therapeutic resistance. Additionally, the review discusses current clinical trials targeting histone modifications in GBM, highlighting successes, limitations, and future perspectives.

## Introduction

Glioblastoma (GBM), classified as a World Health Organization (WHO) grade IV tumor, is the most prevalent primary malignant tumor of the central nervous system (CNS), accounting for about 51.5% of all malignant brain tumors [1, 2]. Globally, an estimated 321,000 new cases of brain and CNS tumors were reported in 2022, with an age-standardized incidence rate (ASIR) of 1.6 per 100,000 population, according to the 2024 WHO/International Agency for Research on Cancer(IARC) GLOBOCAN database [3]. Regional variations exist; in the United States, data from the Central Brain Tumor Registry of the United States (CBTRUS) indicate a GBM ASIR of approximately 3.23 per 100,000 population between 2017 and 2021 [2]. Similarly, Canadian statistics report a marginally higher GBM incidence of 4.06 per 100,000 population [4], while recent reports from China show an ASIR of 4.17 per 100,000 population in 2022 [5].

Although GBM incidence is lower than that of lung, breast, and colorectal cancers, it is a highly aggressive tumor characterized by frequent recurrences and short survival periods [2, 3]. The standard treatment, referred to as the Stupp regimen, involves maximal safe surgical resection followed by postoperative radiotherapy and chemotherapy and adjuvant temozolomide (TMZ) [6]. Despite these interventions, the overall survival (OS) of GBM patients typically ranges from 12 to 15 months [6, 7]. TMZ sensitivity is linked to the methylation status of the O⁶-methylguanine-DNA methyltransferase (MGMT) gene promoter, with unmethylated MGMT patients showing TMZ resistance and worst outcomes [8]. The EF-14 trial demonstrated that combining tumor-treating fields with the Stupp regimen extends OS to 20.9 months; however, high costs limit its accessibility [9]. Targeted therapies such as bevacizumab, anlotinib, and regorafenib have exhibited limited efficacy [10–12]. Likewise, immune checkpoint inhibitors (pembrolizumab, nivolumab) and peptide vaccines targeting epidermal growth factor receptor (EGFR) variant III (rindopepimut) have failed to demonstrate efficacy in phase II and III trials [13–15]. Meanwhile, oncolytic virotherapy, a potential activator of antitumor immune responses, remains in early clinical development for GBM treatment [16]. Overall, progress in GBM treatment has been slow, highlighting the need for continuous exploration of novel therapeutic strategies to improve patient outcomes. As understanding of GBM molecular mechanisms deepens, emerging approaches targeting epigenetic modifications show promise.

Epigenetic modifications affect gene expression through mechanisms that do not alter the DNA sequence, with histone modifications representing a particularly important category [17–20]. Modifications of core histones (H2 A, H2B, H3, H4), including acetylation and methylation, play critical roles in regulating chromatin structure and gene expression, thereby influencing diverse biological processes [21]. A variety of histone modifications have been identified, including acetylation, methylation, succinylation, crotonylation, and lactylation [22]. Histone modifications are reversible covalent changes, whose deposition, removal, and function are primarily controlled by histone-modifying enzymes and their cofactors [23]. These enzymes are categorized as writers, erasers, and readers. Writers, such as histone acetyltransferases (HATs) and histone methyltransferases (HMTs), catalyze the addition of chemical groups to histones [23]. “Erasers”, including histone deacetylases (HDACs) and histone demethylases (HDMs), remove these modifications. Readers are specialized protein modules that selectively recognize and bind to modified histone residues, thereby facilitating downstream chromatin remodeling events and modulating gene transcriptional regulation [23]. Aberrant histone modifications contribute to disease development and progression, including cancer. Recent studies emphasize their significant roles in the pathogenesis and prognosis of cancers such as gliomas, lymphomas, and prostate cancer [23, 24].

Small-molecule inhibitors targeting histone modifications, such as histone deacetylase inhibitors (HDACis) including vorinostat and romidepsin, are US Food and Drug Administration (FDA) -approved for-approved for cutaneous T-cell lymphoma, with romidepsin and belinostat also approved for peripheral T-cell lymphoma [25, 26]. Subtype-selective HDACi, such as chidamide, shows immunomodulatory synergy in diffuse large B-cell lymphoma by upregulating CD20 expression, thereby enhancing rituximab efficacy [27]. Recent studies highlight that HDACi can reverse hypoacetylation in GBM, restoring tumor suppressor gene expression and enhancing TMZ sensitivity [28]. Tazemetostat, the first oral enhancer of zeste homolog 2 (EZH2) inhibitor, has been approved for EZH2 mutation-positive follicular lymphoma and epithelioid sarcoma [29, 30]. Although currently primarily used for hematologic malignancies, these targeted therapies hold promise for solid tumors as well. For example, HDACi can reverse therapy resistance in GBM by normalizing histone acetylation patterns, whereas EZH2 inhibitors may potentiate immunotherapy efficacy in glioma through immune modulation [28, 31].

**Fig. 1:**
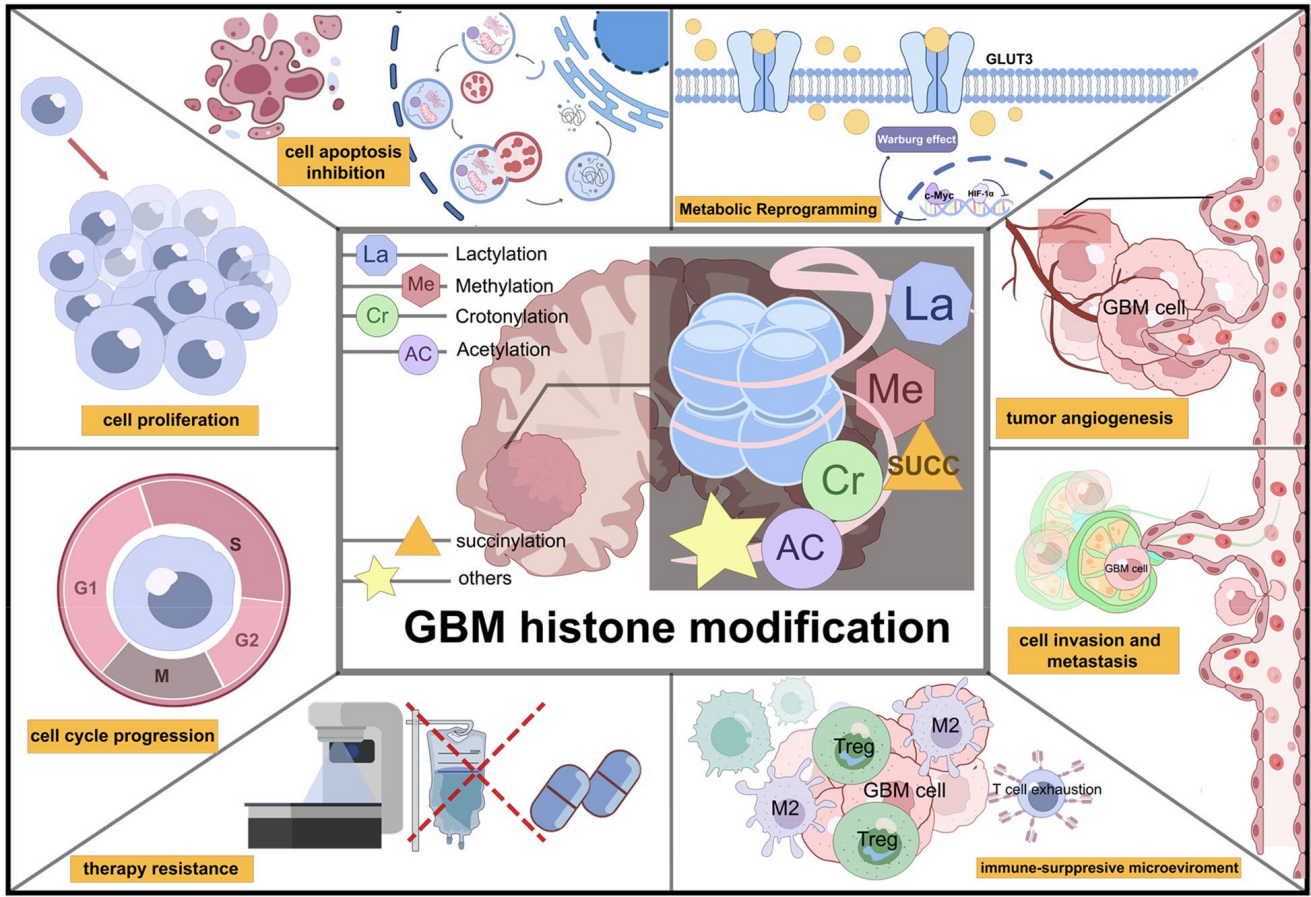
Schematic overview of histone modifications and their regulatory roles in diverse biological processes in GBM. This schematic presents an overview of the main types of histone modifications identified in GBM and the key biological processes they regulate, including proliferation, cell cycle progression, apoptosis, migration, angiogenesis, metabolic reprogramming, therapeutic resistance, and the development of an immunosuppressive microenvironment. For abbreviations, see the abbreviations list

This review discusses their impact on GBM progression, tumor microenvironment remodeling, treatment resistance, and other relevant biological processes (Fig. 1). Furthermore, it summarizes the latest preclinical and clinical advances in targeting histone modifications, offering novel insights to inform future GBM therapies.

## Preclinical studies targeting histone acetylation in GBM

Histone acetylation in GBM is predominantly regulated by histone HATs, which catalyze the addition of acetyl groups to lysine residues, thereby enhancing chromatin accessibility and facilitating gene transcription [21, 32]. Conversely, HDACs remove these acetyl groups, repressing transcription by tightening chromatin structure [33]. Proteins known as readers specifically recognize histone acetylation marks, thereby mediating chromatin remodeling and regulating gene expression [33]. Dysregulation of these acetylation modifiers has been shown to be closely linked to GBM progression and treatment resistance, highlighting them as promising therapeutic targets [34]. These acetylation regulators and readers are further detailed in Table 1.

**Table 1:** The effects of histone acetylation regulators and readers in GBM

Catagory	Enzyme	Effects in GBM	Reference
**Histone** **acetyltransferases** **(writer)**	p300	Inhibition of p300 enhances GBM cell invasion and increases sensitivity to TMZ	[36, 37, 40]
	CBP	Inhibition of CBP suppresses cell proliferation, induces apoptosis, inhibits tumor growth in vivo, and prolongs survival in GBM mouse models	[45, 246]
	PCAF	Knockdown of PCAF is associated with the inhibition of cell proliferation	[49]
	Ti60	Knockdown of Ti60 is associated with the inhibition of proliferation, migration, colony formation, and tumor growth in vivo	[51]
	MOZ(KAT6 A)	Induce apoptosis and suppresses tumor growth in vivo	[54]
**Histone** **deacetylases** **(erasers)**	**Class I**		
	HDAC1	Inhibition of HDAC1 suppresses cell proliferation, invasion, and EMT, reduces stemness markers, induces apoptosis, and inhibits tumor growth in vivo	[64–69]
	HDAC2	Silencing of HDAC2 suppresses cell proliferation, migration, invasion, and stemness, enhances TMZ sensitivity, and reduces glucose uptake levels	[70–73, 247]
	HDAC3	Blockade of HDAC3 inhibits the proliferation and self-renewal of GSCs and induces their differentiation into astrocytes	[76, 77]
	HDAC8	Inhibition of HDAC8 increases DNA damage, decreases cell viability, inhibits cell proliferation, and induces cell cycle arrest and apoptosis	[256]
	**Class II**		
	HDAC4	Knockdown of HDAC4 suppresses cell proliferation, invasion, and tumor growth in vivo, enhances sensitivity to radiotherapy, and induces autophagy	[264, 265]
	HDAC5	Promote U87 cell proliferation and decreases glycolysis and resistance to TMZ in U251 cells	[78, 79]
	HDAC6	Inhibition of HDAC6 reduces GBM cell proliferation, migration, and colony formation, induces apoptosis, decreases autophagy, increases sensitivity to TMZ and radiotherapy, inhibits tumor growth in vivo, and extends survival in mice	[81–86, 249–254]
	HDAC7	Knockdown of HDAC7 inhibits tumor angiogenesis and growth in vivo	[92]
	HDAC9	Knockdown of HDAC9 inhibits cell proliferation and tumor growth in vivo	[93]
	**Class III**		
	Sirt1	Inhibition of Sirt1 has controversial effects; it stimulates proliferation and metastasis, while down-regulation suppresses cell proliferation, induces apoptosis, inhibits tumor growth in vivo, and enhances sensitivity to radiotherapy	[94–98]
	Sirt2	Controversial: Sirt2 overexpression reduces cell proliferation and colony formation while knockdown induces growth arrest and apoptosis of GBM cells, reducing their tumorigenicity	[105–107]
	Sirt3	Down-regulation of Sirt3 inhibits GBM cell proliferation, migration, and glycolysis, induces apoptosis, and suppresses tumor growth in vivo	[111–114]
	Sirt4	Regulate glutamate metabolism to prevent excitotoxicity in GBM cells	[116]
	Sirt6	Suppression of cell growth, induction of apoptosis, and inhibition of the Warburg effect	[118–122]
	Sirt7	Promote cell proliferation and invasion	[123]
	**Class IV**		
	HDAC11	The function remains unknown	
**Acetyllysine** **binding proteins** **(readers)**	BRD2	The inhibition of BRD2 reduces the invasiveness of GBM	[137]
	BDR4	The inhibition of BDR4 suppresses the proliferation of GBM cells, induces apoptosis, and cell cycle arrest, and inhibites tumor growth in vivo	[139–143]

Abbreviations: *GBM* glioblastoma, *TMZ* temozolomide, *EMT* epithelial-mesenchymal transition, *GSCs* glioblastoma stem cells. Note: Only common abbreviations are explained below. Other gene/protein abbreviations can be found in the abbreviation list

### Targeting HATs

**Fig. 2:**
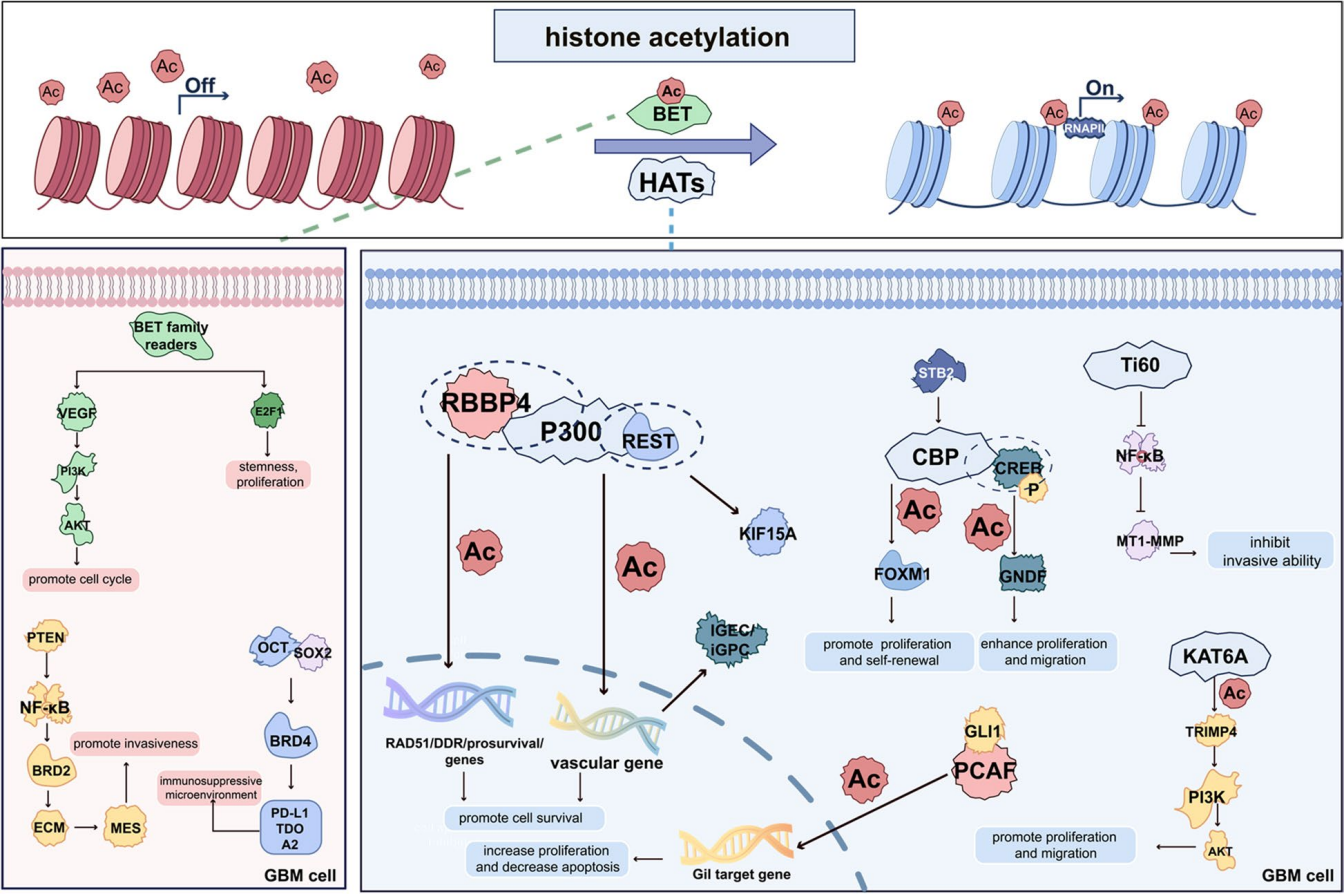
Regulatory functions of histone acetylation–associated readers and writers in GBM. Histone acetylation readers (BET family) modulate GBM biological behaviors by interacting with multiple signaling pathways, such as PI3 K/AKT and MEK/ERK, and by influencing the tumor immune microenvironment (on the left side of the figure). Various HATs affect GBM cell proliferation, invasion, migration, apoptosis, and angiogenesis through different molecular pathways, including NF-κB and PI3 K/AKT (on the right side of the figure). For clarity, only major regulatory axes are depicted; further mechanistic details are discussed in the main text. For abbreviations, see the abbreviations list

HATs are classified into three major families based on structural and functional characteristics: the orphan family, including E1A binding protein p300 (p300) and CREB-binding protein (CBP); the Gcn5-related N-acetyltransferase (GNAT) family such as p300/CBP-associated factor (PCAF, also known as KAT2B); and the MYST family, which includes monocytic leukemia zinc finger protein (MOZ), Ybf2, SAS2, and Tat-interacting protein 60 (Tip60) [33] (Fig. 2).

Among them, p300 and CBP share significant homology and are considered critical in GBM. Specifically, p300 promotes vascular gene expression in glioblastoma stem cells (GSCs) under radiation stress via H3 K27 acetylation, facilitating their transdifferentiation into vascular endothelial-like and pericyte-like cells. Inhibition of p300 by shRNA or small molecules like C646 has been shown to reverse this process and suppress tumor growth [35, 36]. Furthermore, p300 forms complexes with key factors such as repressor element-1 silencing transcription factor (REST) and retinoblastoma-binding protein 4 (RBBP4), which regulate oncogene transcription and DNA damage repair genes to support GBM cell survival [37–40]. The HAT inhibitor BF1 targets p300 and reduces global H3 acetylation; however, its impact on GBM malignancy requires further investigation [41]. Additionally, long non-coding(lnc) TALC (temozolomide-associated lncRNA in glioblastoma recurrence) promotes TMZ resistance by activating the c-Met/signal transducer and activator of transcription 3(STAT3)/p300 axis, which increases H3 acetylation at the MGMT promoter, thereby enhancing MGMT expression [42]. Complex interactions involving p300, such as with SMAD family member 1 (SMAD1) and tumor protein p53 (p53), modify acetylation patterns, which may contribute to tumor growth and chemoresistance [43].

Similarly, CBP is overexpressed in GBM and has been reported to facilitate malignant phenotypes through cooperation with cyclic-AMP response element-binding protein (CREB) to promote glial cell line-derived neurotrophic factor (GDNF) transcription, which enhances proliferation and migration [44, 45]. The dual inhibitor HAT inhibitor II selectively targets p300/CBP, activates the p53 pathway, and induces caspase-dependent apoptosis in GBM cells, suggesting therapeutic potential [46]. The GNAT family member PCAF regulates p53 transcriptional activity and impacts the Hedgehog (Hh) signaling pathway by interacting with glioma-associated oncogene homolog 1 (Gli1). This interaction reduces H3 K9 acetylation on Hh target promoters, modulating gene expression and suppressing GSCs proliferation and tumorigenicity when silenced [47–49]. Hence, PCAF presents a potential target for GBM therapy.

Members of the MYST family, such as Tip60 and MOZ, also influence GBM pathophysiology. In GBM, the low expression of Tip60 inversely correlates with matrix metalloproteinase 14 (MT1-MMP) levels via modulation of the nuclear factor kappa-light-chain-enhancer of activated B cells (NF-κB) pathway and has been shown to inhibit the invasive capacity of GBM cells [50, 51]. MOZ (also known as KAT6 A), involved in gene transcription and cellular senescence, is overexpressed in GBM and has been linked to poor prognosis [52, 53]. Knockdown of KAT6 A reduces H3 K23 acetylation in GBM cells, which has been shown to suppress multiple malignant phenotypes in vitro and subsequently diminish tumor growth in an orthotopic mouse xenograft model system [54]. Mechanistically, KAT6 A promotes tumorigenesis by facilitating the binding of H3 K23ac to the tripartite motif containing 24 (TRIM24), which has been reported to activate the phosphatidylinositol 3-kinase/protein kinase B (PI3 K/AKT) signaling pathway [54].

### Targeting HDACs

**Fig. 3:**
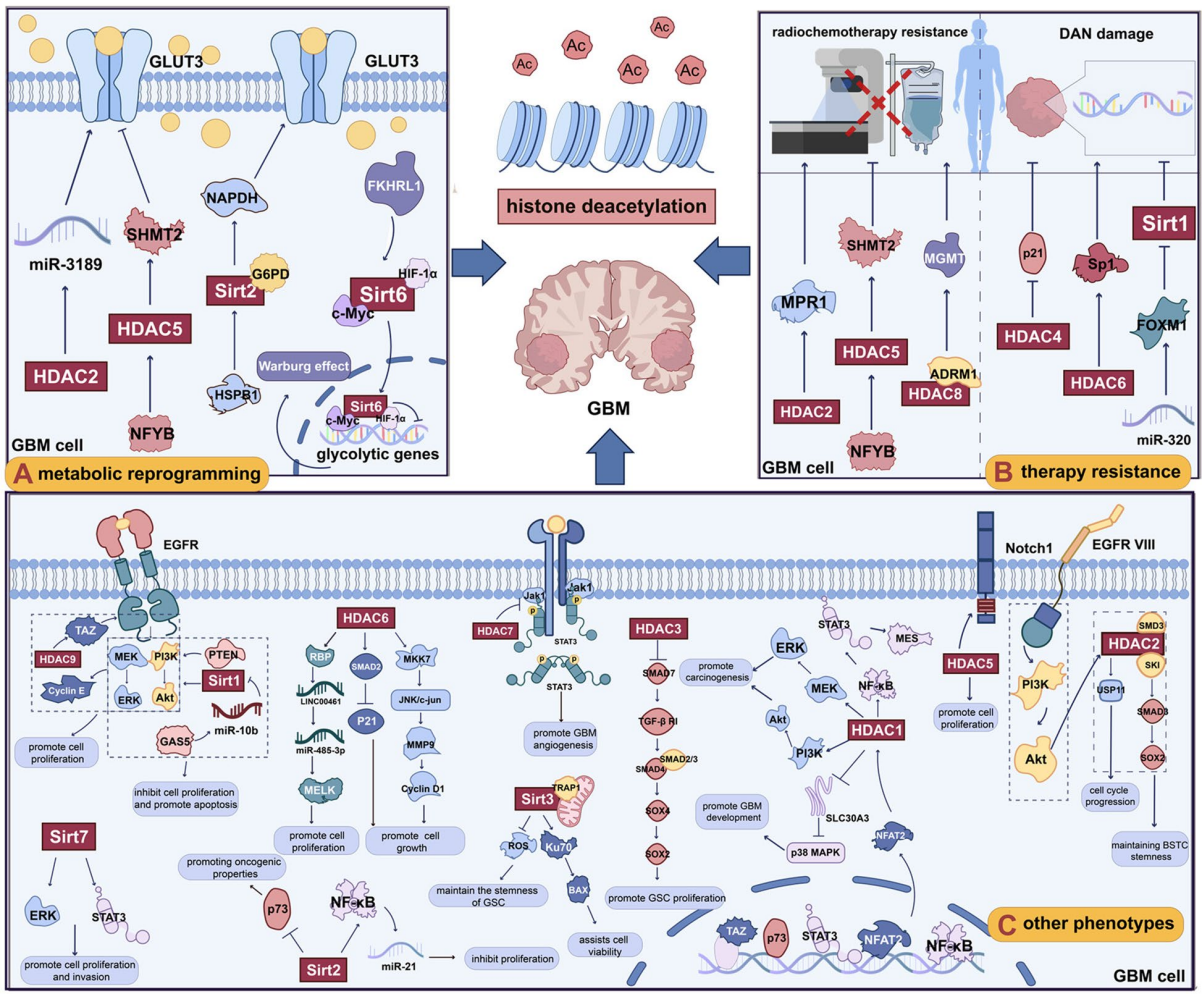
Roles of HDACs in modulating GBM biological behaviors. **A** HDACs regulate metabolic reprogramming in GBM, particularly pathways involved in the Warburg effect. **B** HDACs contribute to chemotherapy and radiotherapy resistance through mechanisms such as modulating MGMT expression and the DNA damage response. **C** HDACs impact GBM cell proliferation, invasion, migration, autophagy, and angiogenesis via multiple signaling pathways, including MEK/ERK, NF-κB, and PI3 K/AKT. For clarity, only representative pathways and functions are shown; detailed mechanisms are provided in the main text. For abbreviations, see the abbreviations list

HDACs are categorized into four primary classes according to structural and sequence homology: class I (HDAC1, HDAC2, HDAC3, HDAC8), class II (HDAC4, HDAC5, HDAC6, HDAC7, HDAC9, HDAC10), class III (Sirtuins 1–7, Sirt1-7), and class IV (HDAC11) [55]. Classes I, II, and IV HDACs depend on zinc ions within their catalytic sites to stabilize acetylated substrates and catalyze deacetylation reactions [56, 57]. Class III HDACs use nicotinamide adenine dinucleotide (NAD⁺) as a co-substrate to catalyze acetyl group removal from histones, converting NAD⁺ into nicotinamide and O-acetyl ADP-ribose [58]. HDACs modulate gene expression by deacetylating lysine residues on histones H3 and H4, as well as modifying non-histone proteins, thereby influencing multiple downstream signaling pathways [59–62]. Studies have shown that HDACs affect the malignant biological processes of GBM through various mechanisms (Fig. 3).

#### Class I HDACs

Elevated HDAC1 has been reported to negatively correlate negatively correlates with patient survival and may promote tumorigenesis by activating the PI3 K/AKT and mitogen-activated protein kinase kinase/extracellular signal-regulated kinase (MEK/ERK) signaling pathways [63–66]. The nuclear factor of activated T cells 2 (NFAT2) regulates HDAC1 expression. HDAC1 deacetylates the NF-κB subunit p65, modulating NF-κB transcriptional activity and potentially promoting the mesenchymal transition in GSCs, which may sustain GSCs malignancy [67]. Knockdown of HDAC1 has been shown to increase H3 K4/19ac and H3 K27ac, which is associated with activation of p53 and reduced expression of stemness-associated genes [66, 67]. In p53 wild-type GSCs, HDAC1 depletion has been found to activate STAT3, which may enhance tumor aggressiveness, suggesting that dual targeting of STAT3 and HDAC1 could serve as a potential therapeutic strategy for p53 wild-type GBM [66]. Moreover, elevated HDAC1 has been shown to promote deacetylation of H3 K27ac at the solute carrier family 30 member 3 (SLC30A3) locus, which is associated with repression of its expression and may facilitate GBM progression through the p38 mitogen-activated protein kinase (MAPK) signaling pathway. Thus, targeting the HDAC1/SLC30A3/p38 MAPK axis may offer therapeutic benefits in GBM [68]. The HDAC1 inhibitor pyroxamide and the HDAC1/3 inhibitor RGFP109 have been reported to suppress epithelial-mesenchymal transition (EMT) and invasion in GBM cells [68, 69].

HDAC2 has been implicated in maintaining the stemness of GSCs [70]. It specifically interacts with transforming growth factor β (TGF-β) pathway protein SMAD3 and Ski proto-oncogene (SKI) to help maintain the tumorigenicity of brain tumor stem cells (BTSCs) both in vitro and situ xenograft models [70]. Treatment with romidepsin, an HDAC2 inhibitor, has been shown to reduce BTSCs growth by increasing H3 and H4 acetylation [70]. Furthermore, HDAC2 participates in the regulation of glucose metabolism in GBM [71, 72]. HDAC2 knockdown represses transcription of glucose transporter 3 (GLUT3) by modulating microRNA-3189 (miR-3189) levels, thereby decreasing glucose uptake and inducing cell death as well as tumor suppression [71]. HDAC2 also regulates cell cycle progression through the EGFRvIII/PI3 K/AKT signaling axis. FK228, an inhibitor targeting both HDAC1 and HDAC2, has been shown to induce cell cycle arrest in GBM cells harboring EGFRvIII mutations [73].

HDAC3 contributes to cell cycle regulation and genome stability and its expression negatively correlates with survival in GBM patients [74, 75]. The HDAC3 inhibitor RGFP966 has been shown to upregulate SMAD7 transcription, thereby inhibiting TGF-β signaling and suppressing proliferation and self-renewal of GSCs [76]. However, RGFP966 has also been shown to activate STAT3 and interleukin-6 (IL-6), indicating that HDAC3 inhibitors may need to be combined with other therapies for optimal efficacy [77].

#### Class II HDACs

HDAC5 expressions may contribute to the heterogeneity of GBM. It is upregulated in GBM cell lines such as U87 and LN-229, where it has been shown to promote proliferation by enhancing neurogenic locus notch homolog protein 1 (Notch1) expression [78]. Additionally, HDAC5 has been reported to be involved in TMZ resistance, which will be discussed further in later sections [79].

HDAC6 is the most extensively studied histone deacetylase in GBM. Multiple studies have confirmed its important role. Elevated HDAC6 expression has been shown to promote GBM cell proliferation via activation of the mitogen-activated protein kinase kinase 7(MKK7)/c-Jun N-terminal kinase (JNK)/c-Jun signaling pathway [80]. Simultaneously, HDAC6 has been shown to downregulate cyclin-dependent kinase inhibitor 1A (p21) expression by inhibiting SMAD2 phosphorylation, thereby attenuating TGF-β receptor signaling and facilitating tumor growth [81]. Furthermore, high-temperature requirement A serine peptidase 1 (HTRA1) may enhance the binding between HDAC6 and α-tubulin by reducing α-tubulin acetylation, promoting GBM cell migration [20]. The inhibition of HDAC6 has been shown to suppress GBM cell proliferation, migration, colony formation, EMT, and autophagy [82, 83]. Additionally, HDAC6 interacts with RNA-binding proteins (RBPs) to stabilize LINC00461 expression. LINC00461 acts as a molecular sponge for the tumor-suppressive microRNA miR-485-3p, maintaining expression of the cell cycle regulator maternal embryonic leucine zipper kinase (MELK) and promoting GBM proliferation. This axis has been shown to be disrupted by the HDAC6 inhibitor MPT0B291 [84]. Therefore, HDAC6 may represent a promising therapeutic target in GBM. Moreover, HDAC6 has been reported to be closely linked to resistance against TMZ chemotherapy and radiotherapy, topics that will be elaborated on in later sections [85, 86]. Several HDAC6 inhibitors are under development. Sahaquines have been shown to inhibit α-tubulin acetylation, significantly reducing GBM cell viability, invasiveness, and relapse risk [87]. The compound J22352 has been shown to markedly suppress GBM malignancy and diminish PD-L1-mediated immunosuppression, potentially enabling combination with immunotherapy [88]. Moreover, selective HDAC6 inhibitors such as JOC1, hydroxamic acid 16, and CAY10603 have demonstrated significant antitumor efficacy in GBM models [89, 90]. Although HDAC6 inhibitors show promise in preclinical studies, further research is needed to advance their clinical translation.

HDAC7 expression correlates with mesenchymal markers in GBM. Knockdown of HDAC7 has been shown to reprogram STAT3 signaling, thereby suppressing angiogenesis and tumor growth [91, 92]. HDAC9 is overexpressed in GBM patients with poor prognosis and has been reported to activate the EGFR pathway through the transcriptional co-activator with PDZ-binding motif (TAZ). This activation stimulates the PI3 K/AKT and ERK pathways, thereby promoting tumor progression. Knockdown of HDAC9 has been shown to suppress these oncogenic phenotypes [93]. Currently, specific inhibitors targeting HDAC7 and HDAC9 in GBM have not been developed. Finally, the role of HDAC10 in GBM remains largely unexplored.

#### Class III HDACs

Studies have shown that Sirt1 is downregulated in GBM tissues and cell lines. Circular RNA 0005075 promotes GBM progression by inhibiting Sirt1, suggesting a potential tumor-suppressive role for Sirt1 [94]. However, other reports indicate Sirt1 is upregulated in GBM, reflecting its possible oncogenic function. Sirt1 has been shown to support GBM cell survival through a p53-dependent mechanism [95, 96]. lncRNA-GAS5 inhibits GBM tumorigenesis by suppressing Sirt1, activating phosphatase and tensin homolog (PTEN), and blocking the PI3 K/AKT and MEK/ERK signaling pathways [97]. Moreover, miR-320 enhances GBM radiosensitivity by targeting forkhead box M1 (FOXM1), thereby reducing Sirt1 expression [98]. Inhibition of casein kinase 2 (CK2), as well as treatment with Sinomenine and betulinic acid derivative B10, has been shown to downregulate Sirt1, leading to enhanced p53 transcriptional activity and apoptosis induction in GBM cells [99, 100]. Collectively, these findings suggest that Sirt1 may exert a pro-oncogenic role in GBM. Sirt1 activators F0911-7667 and SRT2183 have been shown to exert anticancer effects in GBM by inducing autophagic cell death and endoplasmic reticulum stress, respectively [101, 102]. The Sirt1-specific inhibitor EX527 has been shown to activate the p53 pathway, thereby inducing apoptosis [103]. Deoxypodophyllotoxin has been shown to activate the JNK/Sirt1 axis, promoting parthanatos in GBM cells [104]. Hence, the role of Sirt1 in GBM remains controversial, potentially due to diverse regulatory mechanisms.

Similarly, Sirt2 exhibits dual roles in GBM. Sirt2 has been shown to suppress tumor growth by inhibiting proliferation through the NF-κB-miR-21 pathway, while also promoting oncogenesis by repressing transcription of the tumor suppressor p73 [105, 106]. Heat shock protein B1 (HSPB1) facilitates Sirt2-mediated activation of glucose-6-phosphate dehydrogenase (G6PD), thereby promoting nicotinamide adenine dinucleotide phosphate (NADPH) and pentose production in GBM cells, which has been shown to support proliferation [107]. The Sirt2-specific inhibitor AGK2 has been shown to suppress the malignant phenotype of GBM cells [106]. Furthermore, Compound 18 has been shown to effectively inhibit GBM cell growth by targeting both Sirt1 and Sirt2 [108].

Sirt3 is highly expressed in GBM and correlates with poor prognosis. Sirt3 has been shown to promote cell viability through the Ku70-BAX pathway and interact with tumor necrosis factor(TNF) receptor-associated protein 1 (TRAP1) to enhance mitochondrial respiratory capacity and reduce reactive oxygen species (ROS) levels, thus preserving GSCs stemness [109–111]. Inhibition of Sirt3 has been shown to induce metabolic reprogramming, mitochondrial autophagy, and ferroptosis by downregulating solute carrier family 7 member 11 (SLC7A11), a critical ferroptosis antagonist [112]. Atractylon and fraxinellone have been shown to inhibit Sirt3, thereby suppressing GBM progression [113, 114].

Sirt4 has been reported to be critical for maintaining genomic stability and suppressing tumor growth through inhibition of mitochondrial glutamine metabolism [115]. In GBM, Sirt4 has been reported to protect against excitotoxicity-induced cell death by modulating glutamate metabolism [116]. Sirt5 is poorly expressed in GBM and negatively correlates with patient prognosis [117]. Sirt6, regulated by mammalian sterile 20-like kinase 1 (MST1), miR-33, and forkhead in rhabdomyosarcoma-like 1 (FKHRL1, also called FOXO3a), has been shown to inhibit GBM cell growth via multiple mechanisms. These include promoting apoptosis, reducing oxidative stress, suppressing Janus kinase 2 (JAK2)/STAT3 signaling, downregulating Notch3, and decreasing glycolytic activity, thereby limiting the Warburg effect [118–122]. Moreover, FOXO3a binds to the Sirt6 promoter, upregulating its expression, which has been shown to subsequently inhibit cell proliferation and promote apoptosis [122]. Sirt7 has been shown to promote GBM cell activity through activation of the ERK/STAT3 pathway [123].

Although the role of class IV, HDAC11 in GBM remains unclear, its downregulation correlates with patient survival [63]. Table 2 provides a summary of currently known HDAC interactions with related molecules and their roles in GBM.

**Table 2:** Interactions between HDACs and associated molecules and their roles in GBM

HDAC	Direct Interaction Molecular	Molecular Interactions Effect on GBM	Relevant Signaling Pathways	Reference
HDAC1	NFAT2	Enhance HDAC1’s deacetylase activity, promoting NF-κB p65 deacetylation and driving GBM mesenchymal transition	NF-κB signaling pathway	[67]
HDAC2	SMAD3/SKI complex	Maintain brain tumor stem cell tumorigenicity	TGF-β signaling pathway	[70]
HDAC5	NFYB	Suppress SHMT2 expression, thereby reducing glycolysis and TMZ resistance in GBM cells	NFYB-HDAC5-SHMT2	[79]
HDAC6	RBP	Stabilize LINC00461, sponging miR-485-3p and promoting MELK expression and GBM cell proliferation	HDAC6/RBP/LINC00461/miR-485-3p/MELK	[84]
HDAC8	ADRM1	Regulate MGMT protein levels	TMZ resistance	[256]
HDAC9	TAZ	Lead to EGFR phosphorylation, driving GBM progression	PI3 K/AKT and ERK pathways	[93]
Sirt2	HSPB1 and G6PD	HSPB1 facilitates the interaction between G6PD and Sirt2, promoting deacetylation and activation of G6PD, sustaining NADPH and pentose phosphate production in GSC	Regulation of cell proliferation	[107]
Sirt3	TRAP1	Enhance mitochondrial respiratory capacity and reduce ROS production, thereby maintaining GSC stemness	Regulation of mitochondrial respiratory function	[111]
Sirt6	FOXO3a	Positively regulate Sirt6’s expression, thus inhibiting cell proliferation and promoting apoptosis	JAK2/STAT3 and Notch signaling pathways	[122]

*Abbreviations*: *HDACs* histone deacetylases, *GBM* glioblastoma, *TMZ* temozolomid, *MGMT* O⁶-methylguanine-DNA methyltransferase, *GSCs* glioblastoma stem cells, *ROS* reactive oxygen species. Note: Only common abbreviations are explained below. Other gene/protein abbreviations can be found in the abbreviation list

#### pan-HDACis

In addition to the HDACis mentioned above, several non-specific or pan-HDACis have been developed. Entinostat (MS275), a class I HDACi targeting HDAC1, HDAC2, and HDAC3, has been shown to suppress FLICE inhibitory protein expression through the myelocytomatosis oncogene (c-Myc) pathway, thereby inducing apoptosis and inhibiting GBM cell growth [124]. The pan-HDACi vorinostat has been shown to downregulate the AKT-mTOR signaling pathway, promoting autophagy and consequently slowing tumor progression in GSCs [125]. Panobinostat (LBH589) has been shown to disrupt the heat shock protein 90 (Hsp90)/HDAC6 complex and induce degradation of hypoxia-inducible factor 1α (HIF-1α), resulting in decreased vascular endothelial growth factor (VEGF) expression and subsequent inhibition of tumor angiogenesis and growth [126]. Trichostatin A (TSA) has been shown to upregulate p53 and p21 expression, leading to cell cycle arrest and inhibition of tumor growth [127]. Givinostat (ITF2357) has been shown to inhibit proliferation and promote apoptosis in GSCs; however, it concurrently induces autophagy, which counteracts its cytotoxic effects. Autophagy inhibition has been shown to enhance Givinostat-induced cell death, suggesting that combining autophagy inhibitors with HDACis may improve therapeutic efficacy in GBM [128]. Tinostamustine, an alkylating HDACi, combines the DNA-damaging properties of bendamustine with the activity of vorinostat and has been shown to produce enhanced antiproliferative effects and increased radiotherapy sensitivity. Its antitumor mechanisms likely involve inhibition of DNA repair and suppression of radiotherapy-induced autophagy [129]. The HDACi Scriptaid has been shown to induce apoptosis via activation of the JNK pathway, concomitantly decreasing telomerase activity [130].

### Targeting histone acetylation readers

Histone acetylation readers play a crucial role in chromatin remodeling and gene transcription by recognizing acetylated lysines through their N-terminal tandem bromodomains (BRDs). The bromodomain and extra-terminal domain (BET) family, comprising BRD2, BRD3, BRD4, and BRDT, is the most extensively studied group of histone acetylation readers [131]. BET inhibitors, including JQ1, I-BET151, and OTX015, have been shown to exert antitumor effects in GBM by targeting signaling pathways such as VEGF/PI3 K/AKT, and disrupting the coactivation transcriptional program of E2 F1 and BET proteins in GBM cells [132–134]. Recent studies report that elevated BRD4 expression in GBM correlates with poor prognosis. Furthermore, co-expression of octamer-binding transcription factor 4 (Oct4) and sex determining region Y-box 2 (SOX2) induces BRD4/H3 K27 Ac-dependent immunosuppressive genes, including PD-L1 and CD70 [135]. JQ1 has been shown to effectively inhibit this immunosuppressive phenotype, suggesting that BET inhibitors may enhance the efficacy of immunotherapy [135, 136]. BRD2 has been reported to modulate extracellular matrix gene expression and promote mesenchymal transition through the PTEN–NF-κB–BRD2 signaling axis, thereby enhancing GBM cell invasiveness [137]. The BRD2 inhibitor GSK620 has been shown to reduce GBM invasiveness and synergize with TMZ and radiotherapy to improve treatment efficacy [137].

Especially, unlike BRD4 inhibitors, BRD4 degraders are chimeric molecules utilizing proteolysis targeting chimera (PROTAC) technology to selectively degrade BET proteins [138]. PROTACs recruit target proteins to intracellular E3 ubiquitin ligases, inducing ubiquitination and subsequent proteasomal degradation for targeted protein elimination [138]. Several BRD4 degraders, including GNE987, dBET6, ZBC260, and ARV-825, have recently been developed and have been shown to exhibit substantial antitumor efficacy in GBM models. dBET6 effectively induces BET protein degradation and has been reported to demonstrate superior antiproliferative activity and overcome intrinsic and acquired resistance of GBM cells to conventional BET inhibitors [134]. ZBC260 has been shown to suppress tumor growth and stemness in GBM by inhibiting the wingless-related integration site/β-catenin (Wnt/β-catenin) signaling pathway [140]. ARV-825, delivered via nanoparticle-based systems, has been reported to effectively cross the blood–brain barrier, inhibit glioma cell proliferation, induce apoptosis, and modulate the tumor immune microenvironment, highlighting its therapeutic potential in GBM [141]. GNE987 has been shown to demonstrate potent antitumor activity in vitro and subcutaneously transplanted tumor mouse models by targeting oncogenic drivers including c-Myc and S100 A16 [142]. Another BRD4 degrader, MZ1, has been reported to exhibit anti-GBM effects by downregulating syndecan-1 (SDC1) [143]. Collectively, these findings support the potential of PROTAC-mediated BRD4 degradation as a promising targeted therapeutic strategy in GBM.

## Preclinical studies targeting histone methylation in GBM

**Fig. 4:**
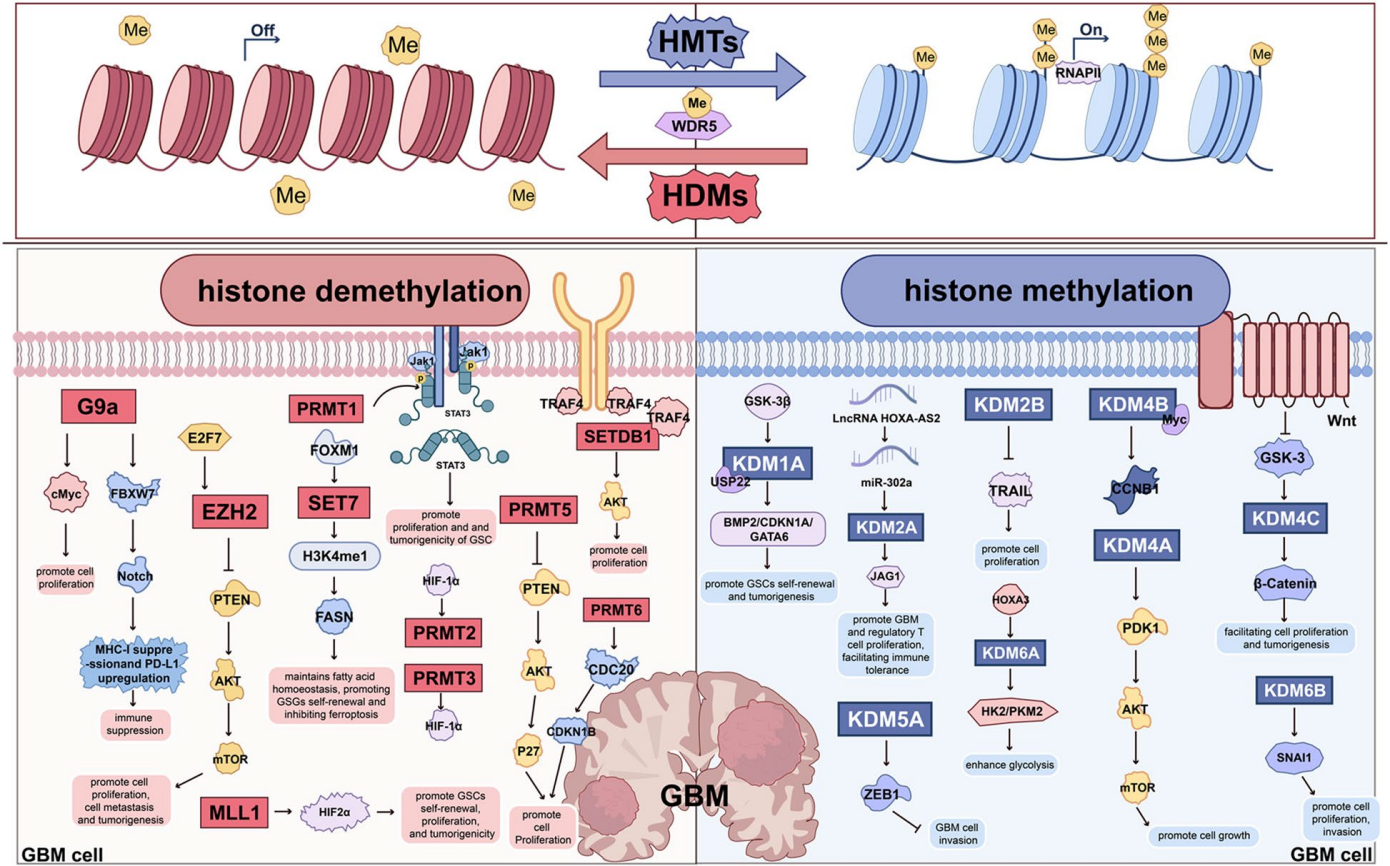
Regulatory roles of HDMs and HMTs in GBM. These enzymes modulate GBM cell behavior through various signaling pathways, such as AKT/mTOR and PTEN, affecting proliferation, invasion, cell survival, and other malignant traits. For clarity, only key mechanisms are illustrated; further details are available in the main text. For abbreviations, see the abbreviations list

Histone methylation primarily occurs on lysine and arginine residues. Lysine methylation exists in mono- (me1), di- (me2), or trimethylated (me3) forms, whereas arginine methylation involves monomethylation and asymmetric dimethylation [144]. These methylation states correspond to distinct biological functions; for instance, H3 K4 me3 is generally associated with gene activation, while H3 K27 me3 correlates with gene silencing [145, 146]. Notably, H3 K27 me3 has been recognized as a crucial epigenetic repressive mark in diffuse midline gliomas and is thought to play an important role in tumor initiation and progression. It has been reported to mediate gene silencing, sustain cellular differentiation, and suppress malignant transformation [146]. According to the 5 th edition of the WHO Classification of Tumors of the CNS, the H3 K27M serves as a key diagnostic marker for diffuse midline gliomas [147]. Enzymes regulating H3 K27 me3 modification, including EZH2, have emerged as potential therapeutic targets. Specific inhibitors are being developed to restore or modulate H3 K27 me3 levels to suppress tumor progression and improve clinical outcomes [146, 148]. In addition, aberrant histone methylation has been shown to be closely associated with the pathogenesis of various cancers, such as breast, prostate, leukemia, and cervical cancers [149–152]. In GBM, histone methylation is also recognized as a key regulatory mechanism (Fig. 4).

### Targeting HMTs

HMTs catalyze the transfer of methyl groups from S-adenosylmethionine to specific histone residues. They are generally classified into lysine methyltransferases (KMTs) and protein arginine methyltransferases (PRMTs). KMTs, typically defined by the presence of SET domains that facilitate methyltransferase activity, include enzymes such as euchromatic histone lysine methyltransferase 2 (EHMT2, G9a), suppressor of variegation 3–9 homolog 1 (SUV39H1), and EZH family members. The PRMT family comprises nine members, designated PRMT1 through PRMT9 [144].

#### KMTs

G9a predominantly catalyzes mono- and dimethylation of H3 K9 and partial methylation of H3 K27 [153]. It is frequently overexpressed in GBM, where its upregulation has been reported to promote tumor cell proliferation, modulate the epigenetic landscape, and disrupt normal cell cycle regulation, thus fostering a pro-tumorigenic microenvironment. The inhibition of G9a has been shown to suppress cellular proliferation, induce cell cycle arrest and apoptosis, activate autophagy by transcriptionally modulating c-Myc, and ultimately diminish tumorigenic potential [154]. Conversely, G9a-mediated dimethylation of H3 K9 has been reported to silence stemness-associated genes such as CD133 and SOX2, impairing GSCs self-renewal capacity [155]. Furthermore, G9a and G9a-like protein (GLP) have been shown to directly catalyze the methylation of HIF-1α, which may inhibit its transcriptional activity and downstream gene expression, thereby reducing the migratory capacity of GBM cells under hypoxic conditions [156]. The G9a-specific inhibitor BIX-01294 has been shown to suppress GBM cell proliferation and malignant phenotypes by inhibiting H3 K9 methylation. Additionally, BIX-01294 has been shown to increase tumor cell sensitivity to TMZ via upregulation of autophagy- and differentiation-associated genes [157–159]. Recent research suggests that G9a may promote GSCs stemness and an immunosuppressive tumor microenvironment by catalyzing H3 K9 me2 at the promoter of F-box and WD repeat domain-containing 7 (FBXW7), repressing its transcription and activating Notch signaling, which can lead to MHC-I suppression and PD-L1 upregulation. Targeting G9a in murine models has been shown to reduce stemness, reactivate anti-tumor immunity, and inhibit tumor growth [160].

Polycomb repressive complex 2 (PRC2) is part of the polycomb group family of epigenetic regulators. EZH2, the catalytic subunit of PRC2, catalyzes trimethylation of histone H3 lysine 27 and is frequently upregulated in GBM, which has been associated with poor prognosis [161, 162]. miR-101 has been reported to upregulate EZH2 and thereby promote tumor growth, invasion, angiogenesis, and chemoresistance, whereas miR-137 and miR-138 downregulate EZH2, suppressing the malignant phenotype [163–166]. Additionally, lncRNA H19 has been shown to interact with EZH2 to repress naked cuticle homolog 1 (NKD1) transcription via H3 K27 me3, which may enhance cell viability and invasion [167]. LncRNA AGAP2-AS1 has been shown to recruit EZH2 to repress the transcription of tissue factor pathway inhibitor 2 (TFPI2), which could contribute to oncogenesis [168]. SOX9 has been suggested to upregulate lncRNA PXN-AS, facilitating EZH2 recruitment to the dickkopf-1 (DKK1) promoter, inducing methylation, and potentially promoting tumorigenesis [169]. EGFR may upregulate lncRNA NEAT1, which interacts with EZH2 to activate the Wnt/β-catenin pathway and thereby facilitate malignant progression [170]. Thus, miRNAs and lncRNAs are thought to play important roles in modulating EZH2 activity in GBM. Additionally, E2F transcription factor 7 (E2F7) has been reported to interact with EZH2 to repress PTEN, which may activate the AKT/mammalian target of rapamycin (mTOR) pathway and promote proliferation and tumorigenesis [171]. A recent study suggests that the canonical NF-κB pathway may activate EZH2 transcription, driving H3 K27 me3 reprogramming and contributing to malignant progression in GBM [172]. Furthermore, EZH2 may promote GBM progression through the ELL-associated factor 2 (EAF2)-HIF-1α and miR-9/Twist signaling axes [173]. EZH2 has been considered essential for maintaining GSCs stemness. Phosphorylation of EZH2 has been shown to activate STAT3 signaling, which may enhance GSCs tumorigenicity [168]. It may also sustain GSCs stemness by directly regulating c-Myc transcription and modulate their proliferation and self-renewal via the MELK-FOXM1-EZH2 axis, potentially protecting GSCs from radiation-induced apoptosis [174]. Moreover, EZH2 has been shown to interact with heterochromatin protein 1 binding protein 3 (HP1BP3) in GSCs to activate WNT family member 7B (WNT7B) expression, which may contribute to TMZ resistance and GSCs stemness [175]. EZH2 inhibitors, including 3-deazaneplanocin A (DZNep) and GSK126, have been shown to attenuate the malignant phenotype of GBM cells [164, 177]. Melatonin has been shown to inhibit GSCs viability and self-renewal by suppressing the EZH2-Notch1 signaling axis [178]. GSK343, a novel EZH2 inhibitor, has been shown to regulate apoptosis and autophagy by modulating canonical and non-canonical NF-κB/IκBα pathways and may demonstrate efficacy in inhibiting GBM cell proliferation in vitro and tumor growth in subcutaneously transplanted tumor mouse models [179]. Collectively, these findings suggest that EZH2 may represent a promising therapeutic target in GBM.

Besides the well-studied G9a and EZH2, other HMTs may be involved in GBM pathogenesis but remain less explored. Mixed-lineage leukemia 1 (MLL1, KAT2A) has been shown to directly methylate H3 lysine 4 and is highly expressed in GSCs. Inhibition of MLL1 has been reported to decrease hypoxia-inducible factor 2α (HIF-2α) transcription, which may subsequently impair GSCs self-renewal, proliferation, and tumorigenicity [180]. Suv39H1, an H3 K9 methyltransferase, is reported to be overexpressed in GBM, and its inhibition may suppress the proliferation and migration of GBM cells [181]. Recent studies have identified Suv39H1 as a potential regulator of GSCs stemness and tumorigenicity. Knockdown of Suv39H1 has been shown to impair GSCs stemness and proliferation. The compound TCH1036 has been shown to inhibit Suv39H1 expression and suppress GBM cell growth in vitro [182, 183]. Chaetocin, a specific Suv39H1 inhibitor, has been shown to reduce the malignant phenotype of GSCs, enhance their sensitivity to TMZ, and inhibit tumor formation in a patient-derived xenograft model [184]. The SET domain bifurcated histone lysine methyltransferase 1 (SETDB1) catalyzes dimethylation and trimethylation of H3 K9 and has been reported to be overexpressed in GBM. The knockdown of SETDB1 has been shown to suppress malignant phenotypes in GBM [181]. Recent work suggests that TNF receptor-associated factor 4 (TRAF4) interacts with SETDB1, stabilizing it via atypical ubiquitination, which may activate the AKT pathway and promote GBM cell proliferation [185]. SET7, an H3 K4 methyltransferase, has been shown to be transcriptionally upregulated by FOXM1, activating the SET7-H3 K4 me1-fatty acid synthase (FASN) axis. This axis may be essential for initiating fatty acid synthesis in GBM, thereby promoting GSCs self-renewal and inhibiting ferroptosis [186]. Finally, KMT5B has been reported to be downregulated in GBM. Overexpression of KMT5B has been shown to suppress the malignant phenotype of GBM cells; however, its underlying mechanisms require further investigation [187].

#### PRMTs

PRMTs are generally classified into three types based on their catalytic activity: Type I PRMTs (PRMT1, PRMT2, PRMT3, PRMT4, PRMT6, and PRMT8) catalyze asymmetric dimethylation; Type II PRMTs (PRMT5 and PRMT9) catalyze symmetric dimethylation; and Type III PRMT (PRMT7) exclusively catalyzes monomethylation [188]. In GBM, PRMT5 and PRMT6 have been studied most extensively. PRMT5 mediates symmetric dimethylation of histones H3 and H4, modulating chromatin structure and facilitating transcriptional repression [189]. It is overexpressed in GBM and has been reported to correlate with poor prognosis. Knockdown of PRMT5 has been shown to suppress malignant phenotypes in GBM cells and prolong survival in GBM mouse models [190, 191]. Mechanistically, PRMT5 is thought to promote GBM malignancy by modulating signaling pathways including ERK1/2, PTEN/AKT/P27, and through its downstream effector suppressor of tumorigenicity 7 (ST7) [190–192]. Consequently, PRMT5 has emerged as a potential therapeutic target in GBM. Recent studies have demonstrated that inhibition of PRMT5 can disrupt RNA splicing and reduce stemness across the GBM transcriptome. PRMT5 inhibitors, including GSK591 and LLY-283, have been shown to suppress GBM cell proliferation and tumorigenicity. Notably, LLY-283 can cross the blood–brain barrier and has been shown to significantly prolong survival in mouse models, highlighting its potential for clinical application [193]. PRMT6 mainly catalyzes methylation of the second arginine residue on H3 [194]. Its overexpression in GBM has been reported to be negatively correlated with patient prognosis. PRMT6 is thought to regulate GSCs mitosis and tumorigenicity by the methylating regulator of chromosome condensation 1 (RCC1). It also stimulates transcription of cell division cycle 20 (CDC20) through H3R2 me2a, which may facilitate ubiquitination and degradation of cyclin-dependent kinase inhibitor 1B (CDKN1B), thereby promoting GBM cell proliferation [195, 196]. Furthermore, PRMT6 and cyclin-dependent kinase 9 (CDK9) have been reported to co-regulate the expression and activation of YTH N6-methyladenosine RNA binding protein 2 (YTHDF) by promoting degradation of adenomatous polyposis coli (APC) and glycogen synthase kinase 3β (GSK3β), negative regulators of the Wnt/β-catenin pathway, thereby exerting oncogenic effects in vitro and tumor models [197]. PRMT6 also represses transcription of TNF receptor-associated factor 6 (TRAF6), inhibiting ubiquitination and degradation of EZH2 protein, potentially promoting GBM cell invasion and migration [198]. The PRMT6 inhibitor EPZ020411 has been shown to suppress GBM cell invasion and migration in vitro, highlighting PRMT6 as a potential therapeutic target with clinical relevance for GBM patients [197].

PRMT1, PRMT2, and PRMT3 have been reported to be highly expressed in GBM, with PRMT2 and PRMT3 linked to poor patient prognosis. The inhibition of PRMT1 has been reported to suppress the proliferation and tumorigenicity of GSCs by modulating the STAT3 signaling pathway and inducing cell cycle arrest and apoptosis. The PRMT1 inhibitor furamidine has shown therapeutic potential [199]. PRMT2 catalyzes asymmetric dimethylation at histone H3 arginine 8, which is considered essential for maintaining target gene expression. PRMT2 inactivation has been shown to inhibit GBM cell growth and enhance TMZ sensitivity in mouse models, causing significant dysregulation of genes involved in cell cycle progression and oncogenic pathways such as PI3 K/AKT, MAPK, JAK/STAT, and Wnt/β-catenin [200]. Recent evidence suggests that HIF-1α may activate PRMT2 under hypoxic conditions, potentially promoting GBM malignancy. PRMT3 has been reported to facilitate GBM progression by stabilizing HIF-1α and upregulating downstream targets, particularly glycolytic enzymes [201]. The PRMT3 inhibitor SGC707 has been shown to diminish glycolytic capacity and tumor growth in GBM cells [202].

### Targeting KDMs

The KDM family is thought to regulate gene expression by demethylating specific histone lysine residues, demonstrating distinct substrate specificities [203]. KDM1 A has been reported to modulate GBM cellular senescence through regulation of HIF-1α protein stability [204]. Nuclear GSK3β has been shown to promote GSCs self-renewal and tumorigenesis by phosphorylating KDM1 A, which collaborates with ubiquitin-specific protease 22 (USP22) [205]. KDM1 A inhibitors, such as TCP, GSK2879552, OGL002, and SP2509, have been shown to induce cellular senescence by promoting proteasomal degradation of HIF-1α through inhibition of KDM1 A [204]. Novel inhibitors, including NCL-1 and NCD-38, have been reported to reduce GSCs viability and neurosphere formation by activating the unfolded protein response (UPR) pathway, thereby improving survival in GBM mouse models [206]. Activated transcription factor 4 (ATF4) is considered a key regulator of the integrated stress response (ISR) induced by endoplasmic reticulum stress. DDP_38003, a specific KDM1 A inhibitor, has been shown to suppress ATF4 expression, resulting in aberrant ISR and sensitizing GBM cells to stress-induced apoptosis [207]. Another novel inhibitor, 4,5-Dimethoxycanthin-6-one, has been shown to suppress proliferation and induce apoptosis and pyroptosis—a form of pro-inflammatory cell death—in GBM cells, suggesting its potential to activate multiple anti-tumor pathways [208]. Based on the above findings, KDM1 A is thought to serve an oncogenic function in GBM. However, evidence suggests that KDM1 A may exert dose-dependent effects on GBM tumorigenicity. Partial inhibition of KDM1 A has been reported to elevate MYC and stem cell gene expression, potentially promoting tumorigenesis, whereas complete inhibition decreases MYC levels, leading to cell death [209].

KDM2 exists in two isoforms: KDM2A and KDM2B. In GBM, KDM2A regulation is reported to be primarily mediated by microRNAs. miR-3666 and miR-663a have been reported to promote the proliferation, invasion, and stemness of GBM cells by targeting KDM2A [210, 211]. Furthermore, lncRNA HOXA-AS2 has been shown to upregulate KDM2A by suppressing miR-302a, which increases Jagged 1 (JAG1) expression, potentially promoting GBM and regulatory T cell proliferation, facilitating immune tolerance and tumor progression [212]. However, the therapeutic potential of targeting this pathway in GBM remains unclear. KDM2B is reported to be overexpressed in GBM and to regulate the TNF-related apoptosis-inducing ligand (TRAIL) pathway. Knockdown of KDM2B has been shown to induce apoptosis and DNA damage, impair DNA repair, and inhibit GBM cell proliferation [213, 214].

The KDM4 (JMJD2) family, comprising KDM4A, KDM4B, and KDM4C, is reported to be overexpressed in GBM and to mediate demethylation of H3 K9, H3 K36, and histone H1.4 lysine 26 [215]. KDM4A has been shown to promote GBM growth by activating the AKT-mTOR pathway through upregulation of 3-phosphoinositide-dependent protein kinase 1 (PDK1), an effect antagonized by rapamycin, an mTOR inhibitor [216]. KDM4B has been reported to interact with MYC to demethylate H3 K9 me3, thereby activating cyclin B1 (CCNB1) transcription and promoting cell proliferation, suggesting potential for targeting KDM4B in MYC-amplified GBM [217]. In GBM, Wnt signaling has been shown to upregulate KDM4C, enhancing β-catenin transcription and facilitating cell proliferation and tumorigenesis [218]. KDM4C may also promote tumorigenesis by inhibiting p53 and upregulating c-Myc expression. The inhibition of KDM4C has been shown to suppress GBM cell proliferation and tumorigenesis, prolonging survival in mouse xenograft models [218, 219], suggesting KDM4C inhibition as a potential therapeutic strategy for GBM.

The KDM5 family of HMTs is characterized by the presence of JmjC, PHD, and ARID domains, which are thought to confer the ability to demethylate H3 K4, a key process in gene expression regulation [220]. One study reported that KDM5A may demethylate H3 K4, repressing zinc finger E-box binding homeobox 1 (ZEB1) expression, and consequently inhibiting GBM cell invasion and migration in vitro [221]. However, other evidence suggests that KDM5A is overexpressed in GBM and closely associated with TMZ resistance. KDM5A inhibitors, including JIB 04, CPI-455, and GSK-J4, have been shown to inhibit proliferation and induce apoptosis in TMZ-resistant GBM cells [222], indicating a complex, context-dependent role for KDM5A in GBM progression that requires further in vivo studies. Research on KDM5C in GBM remains limited. One study observed elevated expression of KDM5C and HIF-1α at GBM tumor margins [223]. HIF-1α has been reported to upregulate KDM5C, which mediates methylation of genes related to inflammation, stem cell differentiation, and hypoxia response, suggesting KDM5C’s potential involvement in the hypoxic tumor microenvironment and as a therapeutic target [223].

MINA, a JmjC domain-containing histone demethylase, is reported to be overexpressed in GBM and to correlate with poor prognosis [224]. It has been reported to regulate cell cycle progression through demethylation of H3 K9 me3, thereby promoting proliferation and tumorigenesis, indicating its potential as a molecular target for GBM therapy [224].

KDM6A is thought to participate in GBM glucose metabolism and tumor progression. Recent evidence suggests that HOXA3 activates KDM6A transcription, resulting in H3 K27 trimethylation removal on glycolytic gene promoters. This epigenetic regulation may enhance glycolysis and promote GBM growth [225]. The inhibition of KDM6A has been shown to impair DNA repair and increase the radiotherapy sensitivity of GBM cells [226]. In GBM, STAT3 has been reported to negatively regulate KDM6B. STAT3 inhibition leads to the upregulation of KDM6B, which has been shown to suppress GSCs growth and neurosphere formation [227]. Conversely, KDM6B has been reported to upregulate snail family transcriptional repressor 1 (SNAI1), a key EMT factor, thereby promoting GBM cell proliferation, migration, and invasion [228]. Further studies are required to clarify KDM6B’s precise role in GBM.

### Targeting methylation readers

Histone methylation"readers"are protein domains that are thought to specifically recognize methylated lysine or arginine residues on histones, thereby influencing gene expression, chromatin architecture, DNA repair, and various critical biological processes [17]. Domains reported to recognize methylated histone lysines include PHD, chromo, WD40, Tudor, double/tandem Tudor, MBT, Ankyrin repeats, zf-CW, and PWWP domains [229]. Known arginine methylation readers include the ADD domain of DNA methyltransferase Dnmt3a, which contains a PHD motif, as well as the Tudor domain of TDRD3 [229]. WD repeat domain 5 (WDR5), a WD40 domain-containing protein, has been shown to recognize histone methylation and associate with histone methyltransferase complexes to facilitate trimethylation of histone H3 lysine 4 [230]. WDR5 has been reported to be overexpressed in GBM and is associated with poor prognosis. It has been reported to promote GBM cell proliferation and self-renewal by enhancing Myc binding to coactivator-associated arginine methyltransferase 1 (CARM1), thereby upregulating oncogene CARM1 expression [231]. Furthermore, WDR5 is thought to be important for maintaining GSCs identity. Both compound 16, a small molecule WDR5 WIN site inhibitor, and WDR5 knockdown have been shown to reduce expression of genes involved in H3 K4 trimethylation and associated oncogenic pathways, thereby inhibiting GSCs growth and self-renewal in vitro, as well as tumor growth in a flank GBM xenograft model [231, 232]. Chromobox 3 (CBX3), a histone methylation reader, contains a chromodomain that has been reported to specifically recognize di- and trimethylated H3 lysine 9, thereby mediating transcriptional regulation [233]. Studies have suggested that CBX3 may directly repress transcription of Parkin RBR E3 ubiquitin protein ligase (PARK2) and STIP1 homology and U-box containing protein 1 (STUB1) via its chromodomain, reducing ubiquitination and stabilizing EGFR protein levels, thereby promoting GBM initiation and progression [234]. To further elucidate the specific roles of histone methylation in GBM, a comprehensive summary of key findings is presented in Table 3.

**Table 3:** The effects of histone methylation regulators and readers in GBM

Category	Enzyme	Effects in GBM	Reference
**Histone** **methyltransferases** **(writers)**	**KMTs**		
	G9a	Inhibition of G9a suppresses GBM cell proliferation, induces cell cycle arrest, apoptosis, activates autophagy, and promotes tumorigenesis	[154]
	EZH2	Promote GBM cell proliferation, invasion, angiogenesis, and resistance to TMZ, maintain GSCs stemness, and foster tumor growth in vivo	[165, 166, 174–179]
	MLL1	Inhibition of MLL1 reduces self-renewal, growth, and tumorigenicity of GSCs	[180]
	Suv39H1	The inhibition of Suv39H1 suppresses GBM cell proliferation, migration, and colony formation; impairs GSCs proliferation and stemness; enhances GSCs sensitivity to TMZ; inhibits tumor growth in vivo	[182, 183]
	SETDB1	Inhibition of SETDB1 suppresses GBM cell proliferation, migration, and colony formation, and induces apoptosis	[181]
	SET7	Promote self-renewal in GBM and inhibits ferroptosis	[186]
	KMT5B	Overexpression of KMT5B reduces GBM cell proliferation and viability and inhibits tumor growth in nude mice	[187]
	**PRMTs**		
	PRMT1	Promote GBM cell proliferation, invasion, and tumor growth	[199]
	PRMT2	Inactivation of PRMT2 inhibits GBM cell growth and self-renewal of GSCs, and enhances sensitivity to TMZ	[200]
	PRMT3	Promote glycolysis and tumor growth in GBM cells	[202]
	PRMT5	Knockdown of PRMT5 inhibits GBM cell proliferation and invasion, promotes apoptosis, and improves tumor growth and survival rates in xenograft models	[190–193]
	PRMT6	Promote GBM cell proliferation, migration, invasion, EMT, and tumorigenicity of GSCs	[195–199]
**Histone** **demethylases** **(erasers)**	KDM1A	Promote GBM cell proliferation and invasion, enhance self-renewal of GSCs, and contribute to tumorigenesis	[204–206]
	KDM2A	Promote GBM cell proliferation, invasion, and maintenance of stemness, a process regulated by miRNA.	[211, 212]
	KDM2B	The inhibition of KDM2B induces apoptosis and proliferation in GBM cells	[213, 214]
	KDM4A	Promotes GBM cell growth and tumorigenesis in vivo	[216]
	KDM4B	The knockdown of KDM4B inhibits cell survival, proliferation, migration, and invasion	[217]
	KDM4C	Knockdown of KDM4 C significantly inhibits GBM cell proliferation in vitro and tumor formation in vivo, prolonging survival in GBM mouse models	[218, 219]
	KDM5A	Controversial: KDM5 A inhibits GBM cell invasion and migration; inhibition of KDM5 A suppresses the proliferation of TMZ-resistant GBM cells	[221, 222]
	KDM5C	The important factor in the hypoxic microenvironment of GBM	[223]
	MINA	Promote GBM cell proliferation and tumorigenesis	[224]
	KDM6A	Involved in GBM glucose metabolism and tumor progression; inhibition of KDM6 A increases radiation sensitivity in GBM cells	[225, 226]
	KDM6B	Controversial: Overexpression of KDM6B inhibits GSC growth and neurophere formation; promotes GBM cell proliferation, migration, and invasion	[227, 228]
**Methyllysine** **binding proteins** **(readers)**	WDR5	Inhibition of WDR5 disrupts the growth and self-renewal of GSCs in vitro and inhibits tumor growth in vivo	[231, 232]
	CBX3	CBX3 directly represses the transcription of PARK2 and STUB1, stabilizing its expression, and promoting the progression of GBM	[233, 234]

*Abbreviations*: *HDACs* histone deacetylases, *GBM* glioblastoma, *TMZ* temozolomide, *GSCs* glioblastoma stem cells, *EMT* epithelial-mesenchymal transition. Note: Only common abbreviations are explained below. Other gene/protein abbreviations can be found in the abbreviation list

## Targeting other histone modifications

**Fig. 5:**
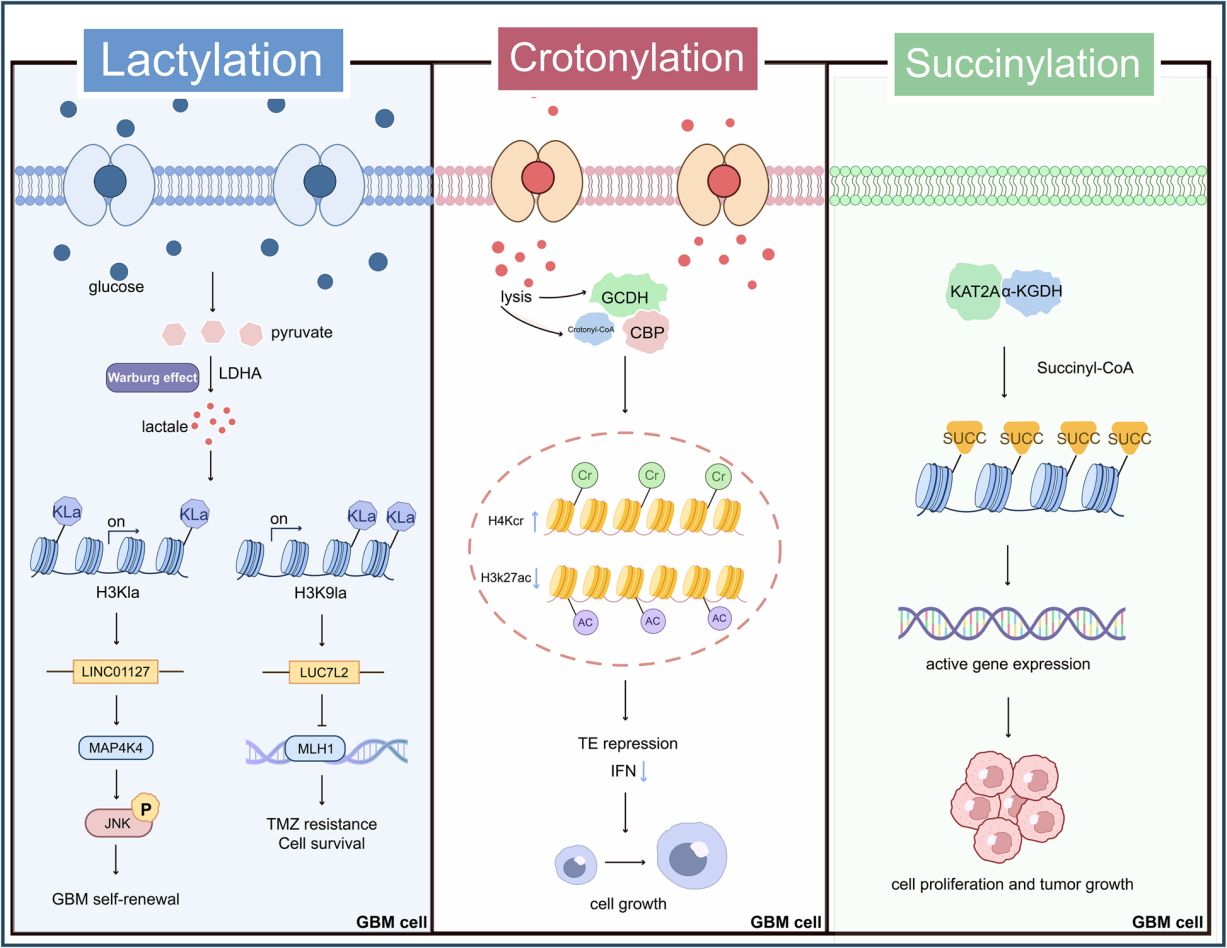
Rare histone modifications and their functions in GBM. Lactylation promotes GBM cell self-renewal, drug resistance, and proliferation via activation of pathways such as NF-κB and MAP4K4/JNK. Crotonylation, elevated by metabolic reprogramming in GSCs, coordinates with other histone modifications to regulate interferon signaling and CD8⁺ T cell infiltration, thereby facilitating tumor growth. Succinylation, catalyzed by KAT2A and α-KGDH, induces H3 K79 succinylation to support GBM cell proliferation and tumorigenesis. Only representative mechanisms are shown; further details are provided in the main text. For abbreviations, see the abbreviations list

In GBM, in addition to common histone acetylation and methylation, rare modifications such as lactylation, crotonylation, and succinylation have been identified [235, 236, 245] (Fig. 5). Histone lactylation, the addition of lactate groups to lysine residues, has been suggested to modulate chromatin dynamics and gene expression, and has been implicated in ocular melanoma, clear cell renal carcinoma, and bevacizumab-resistant colorectal cancer [237–239]. Previous studies have reported that Warburg effect-induced histone H3 lactylation drives the expression of NF-κB-related LINC01127, thereby promoting the self-renewal of GBM cells through the MAP4K4/JNK/NF-κB axis [235]. In GBM, an increased H3 K9 lactylation has been observed in recurrent and TMZ-resistant cells, where it may promote TMZ resistance by upregulating LUC7 like 2 (LUC7L2), a pre-mRNA splicing factor that suppresses MutL homolog 1 (MLH1) and impairs mismatch repair [240]. Notably, the lactate dehydrogenase (LDH) inhibitor stiripentol has been shown to enhance TMZ sensitivity in GBM [240]. Moreover, guanosine triphosphate (GTP)-specific succinyl-CoA synthetase (GTPSCS) has been identified as the first nuclear lactyl-CoA synthetase, catalyzing lactyl-CoA synthesis and reported to cooperate with the acetyltransferase p300 to enhance histone H3 lysine 18 lactylation [241]. This modification has been reported to promote glioma proliferation and radiotherapy resistance by regulating oncogenes such as growth differentiation factor 15 (GDF15) [241]. The therapeutic benefits of targeting this modification in GBM remain unclear, and specific inhibitors against lactylation sites are currently unavailable.

Lysine crotonylation, a recently identified modification, is reported to be dynamically regulated by crotonyltransferases and decrotonylases [242]. Lysine crotonylation levels have been reported to be elevated in colon, lung, thyroid, and pancreatic cancers, where it may influence tumorigenesis [242, 243]. In GBM, GSCs have been reported to reprogram lysine metabolism to increase histone H4 crotonylation via the interaction of glutaryl-CoA dehydrogenase with the crotonyltransferase CBP. This process is thought to modulate H3 K27 acetylation and H3 K9 trimethylation, influencing interferon signaling and CD8 + T cell infiltration, which may promote tumor growth [236]. A lysine-restricted diet combined with anti-PD-1 immunotherapy has been shown to delay tumor progression in GBM [236].

Histone succinylation, the addition of succinyl groups to lysine residues, has been suggested to play a significant role in hepatocellular carcinoma and pancreatic ductal adenocarcinoma. KAT2 A, traditionally known as a HAT, has also been shown to act as a histone succinyltransferase. In GBM, KAT2 A has been reported to associate with the α-ketoglutarate dehydrogenase (α-KGDH) complex, mediating histone H3 lysine 79 succinylation, which may promote cell proliferation and tumorigenesis [244].

## Combination approaches

**Fig. 6:**
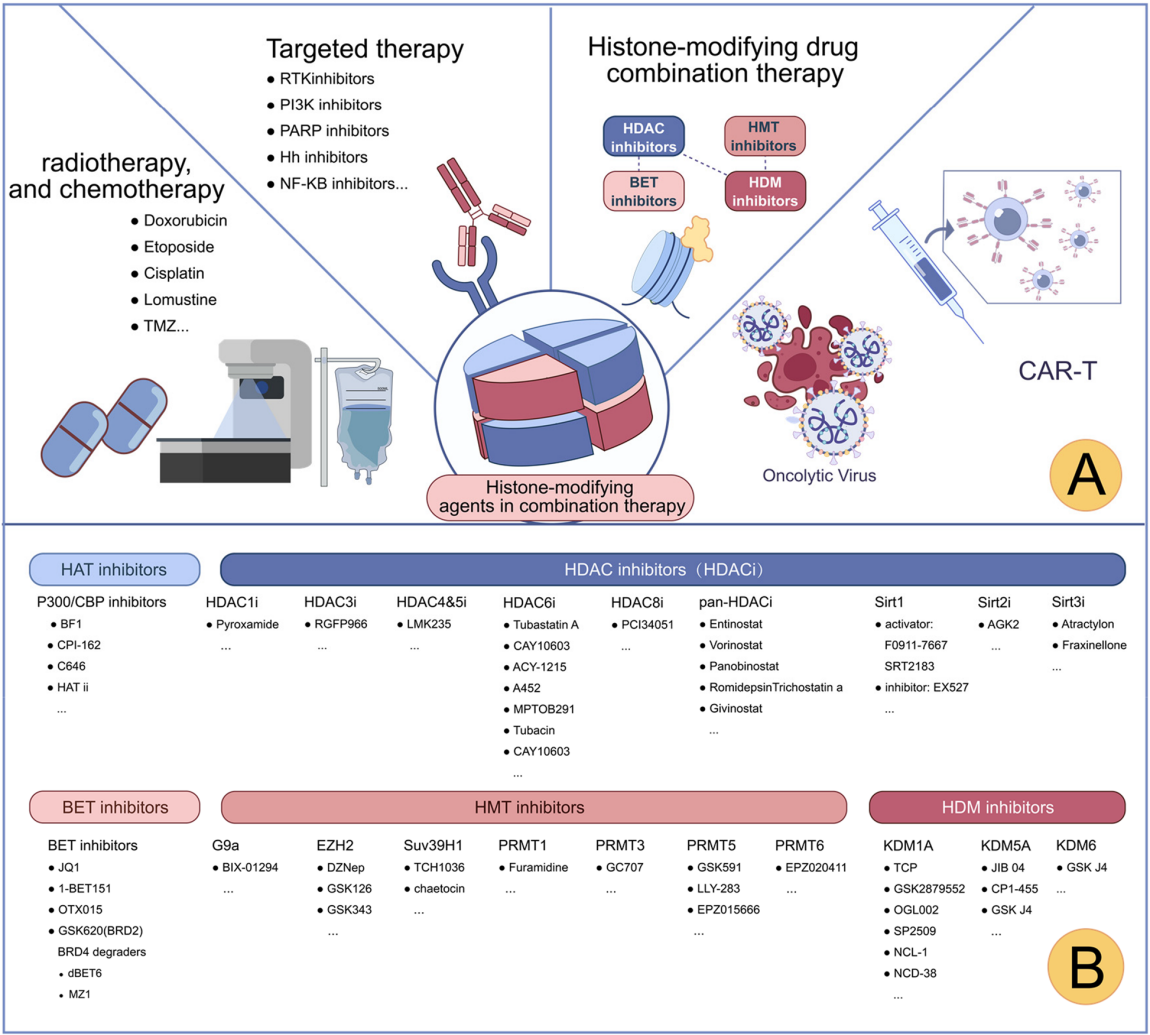
Combination therapy approaches targeting histone modifications in GBM. **A** Schematic of therapeutic strategies combining histone-modifying agents with radiotherapy, chemotherapy, targeted therapy, immunotherapy, and other epigenetic modulators to enhance GBM treatment efficacy. **B** Overview of representative pharmacological agents targeting histone acetylation and methylation in GBM. For abbreviations, see the abbreviations list

GBM is a highly aggressive tumor with a poor prognosis, prompting ongoing research into combination therapies that may improve antitumor efficacy. Advances in the understanding of histone modifications in GBM suggest that combining epigenetic agents targeting these modifications with chemotherapy, radiotherapy, targeted therapy, immunotherapy, or other epigenetic agents may enhance treatment outcomes (Fig. 6). While most evidence remains preclinical, these findings may provide a basis for developing novel therapeutic strategies.

### Overcome chemotherapy and radiotherapy resistance with histone-modifying agents

GBM often exhibits resistance to chemotherapy and radiotherapy, representing an ongoing clinical challenge [28, 245]. Emerging evidence suggests that dynamic histone modifications and epigenetic regulators may play important roles in GBM’s adaptive responses to therapy, potentially presenting novel therapeutic targets.

#### Histone-modifying agents in chemotherapy combinations

P300-related mechanisms, such as lncTALC-mediated activation of the c-Met/STAT3/p300 axis that may contribute to TMZ resistance, and the SMAD1/p53/p300 complex that regulates acetylation to promote tumor growth and chemoresistance, have been previously described [42, 43]. These findings suggest an important role for p300-associated pathways in GBM therapeutic responses. Additionally, Special AT-rich sequence-binding protein 2 (SATB2) has been reported to promote GBM proliferation and self-renewal by recruiting the coactivator CBP to enhance FOXM1 expression [246]. Inhibition of CBP by compounds such as C646 has been shown to increase GBM sensitivity to TMZ and radiotherapy [246]. HDACs are also thought to modulate therapeutic resistance through various pathways. HDAC2 has been reported to sustain GSCs stemness and upregulate multidrug resistance protein 1 (MRP1), facilitating TMZ efflux, while HDAC2 inhibition may reverse this resistance [247]. Although expressed at low levels in GBM, HDAC5 can reportedly be transcriptionally activated by nuclear transcription factor Y subunit β (NFYB), suppressing oncogene serine hydroxymethyltransferase 2 (SHMT2), which may reduce glycolysis and TMZ resistance [79]. LMK235, an HDAC4/5 inhibitor, has been shown to reduce TMZ-resistant GBM cell viability and induce cell autophagy, possibly through downregulation of the oncogene sodium channel epithelial 1α subunit (SCNN1 A) [248]. HDAC6 has been implicated in dual resistance mechanisms. Since EGFR is believed to be important for TMZ resistance, HDAC6 has been shown to regulate EGFR endocytosis and degradation [249, 250]. Studies have reported an increased HDAC6 and EGFR expression in TMZ-resistant GBM cells. Selective HDAC6 inhibitors, including Tubastatin A, CAY10603, ACY-1215, and A452, have been shown to inhibit activation of EGFR and p53 pathways, reduce MGMT expression, and sensitize GBM cells to TMZ [85, 251]. Autophagy is thought to serve as a protective mechanism in GBM, and its inhibition may improve treatment efficacy. Studies suggest that the HDAC6-specific inhibitor Tubacin can inhibit autophagosome formation and autophagosome-lysosome fusion, thereby reducing GBM growth and TMZ resistance in vitro [252]. Furthermore, specificity protein 1 (Sp1), a stress-responsive transcription factor, has been reported to promote stemness and antioxidant gene expression. HDAC1, HDAC2, and HDAC6-mediated deacetylation of Sp1 has been reported to enhance cell proliferation and stem cell maintenance, which may contribute to tumor progression and TMZ resistance [253]. Elevated cytochrome P450 17 A1 (CYP17 A1) expression in TMZ-resistant cells has been reported to be regulated by Sp1; the Sp1-CYP17 A1-DHEA axis is thought to promote TMZ resistance. Concurrent inhibition of CYP17 A1 and HDAC6 may enhance antitumor efficacy [254]. Compound 12, a dual CYP17 A1/HDAC6 inhibitor, has been shown to induce DNA damage and ROS generation, potentially overcoming TMZ resistance in GBM [255]. The HDAC1/2/6 inhibitor MPT0B291 has been reported to enhance Sp1 acetylation, increase TMZ sensitivity in GBM cells, and prolong survival in drug-resistant orthotopic transplantation models [253]. In GBM, HDAC8 has been reported to interact with adhesion regulating molecule 1 (ADRM1) to enhance MGMT expression. HDAC8 inhibitors PCI34051 have been shown to decrease MGMT levels, increase DNA damage, reduce cell viability, and overcome TMZ resistance [256]. Pan-HDACis such as RGFP109, romidepsin, and abexinostat, when combined with TMZ, have been shown to further increase cytotoxicity in preclinical studies [257–259].

Additional epigenetic modifiers have also been implicated in TMZ resistance. EZH2 has been reported to interact with HP1BP3 in GSCs to activate WNT7B expression, which may promote TMZ resistance and GSCs stemness [176]. KDM1A overexpression has been shown to impair DNA repair, whereas KDM1A inhibitors may enhance TMZ efficacy and survival in GBM orthotopic murine models [260]. KDM5A has been reported to be upregulated in TMZ-resistant GBM cells; its inhibition may restore drug sensitivity and suppress tumor growth [222]. BET inhibitors OTX015 and JQ1 have been shown to synergize with TMZ to potentiate antitumor effects [133, 261]. Histone lactylation has also been implicated in TMZ resistance. Stiripentol has been shown to enhance TMZ sensitivity in GBM by inhibiting histone lactylation, suggesting a potentially promising combinational therapy for clinical application [235]. As mentioned above, the Suv39H1 inhibitor Chaetocin has been shown to disrupt the maintenance of GSCs and sensitize them to TMZ [184].

Moreover, HDACis have been shown to synergize with various chemotherapeutic agents. For example, entinostat has been reported to enhance apoptosis when combined with doxorubicin, etoposide, or cisplatin, but decrease apoptosis when combined with vincristine or paclitaxel [261]. Combined treatment with TSA and lomustine has been shown to increase DNA double-strand breaks and enhance lomustine sensitivity, while the KDM6A/B inhibitor GSK-J4 has been reported to sensitize GBM cells to lomustine and etoposide [262, 263].

#### Histone-modifying agents in radiotherapy combinations

Epigenetic plasticity has been suggested to influence radiotherapy resistance in GBM, involving multiple regulators that may modulate DNA repair and autophagy pathways. HDAC6 has been reported to promote DNA repair in GSCs through the sonic hedgehog (SHH)/Gli1/checkpoint kinase 1 (CHK1) signaling axis, and its inhibition by Tubacin may radiosensitize GBM tumors by exploiting this vulnerability [86]. Likewise, HDAC4 knockdown has been shown to upregulate p21, which may impair DNA repair and enhance radiotherapy sensitivity [264, 265]**.** Sirt1 is also related to radiosensitivity [98]**.** Alkylated HDACis like tinostamustine, which merges DNA-damaging bendamustine with HDACi vorinostat, have been reported to exhibit enhanced antiproliferative and radiosensitizing effects, possibly via suppression of radiotherapy-induced autophagy and impaired DNA repair [129]. Panobinostat has been shown to enhance the cytotoxicity of radiation combined with TMZ in GBM cell lines, whereas valproic acid (VPA) has demonstrated radiosensitizing properties in tumor models [266, 267]. In vitro, comparisons have reported radiosensitization rates of ~ 50% for vorinostat, 45% for panobinostat, 40% for VPA, and 60% for entinostat, with optimal effects observed when HDACis are administered 24–48 h before radiotherapy [268]. Beyond HDACs, BRD2 inhibition by GSK620 has been shown to decrease tumor invasiveness and synergize with TMZ and radiotherapy, which may enhance therapeutic responses [137]. Furthermore, PRMT6 is required for methylation of the regulator of RCC1. Its selective inhibitor, EPZ020411, has been shown to block RCC1 arginine methylation, potentially enhancing radiotherapy-induced cytotoxicity in GSC xenograft models [195].

### Histone-modifying agents in targeted therapy combinations

GBM is associated with various oncogenic pathways, including PI3 K/mTOR, MAPK, Hh, and NF-κB signaling. Studies suggest that combining HDACis with agents targeting these pathways may yield synergistic antitumor effects in GBM. For example, the dual PI3 K/mTOR inhibitor dactolisib (BEZ235) with panobinostat, as well as the EGFR inhibitor AG1478 alongside sodium butyrate, have both been shown to reduce GBM cell viability and proliferation [269, 270]. A similar enhancement of cytotoxicity has been observed when 4-phenylbutyrate (4-PB), an HDAC inhibitor, is combined with receptor tyrosine kinase inhibitors such as gefitinib or vandetanib [271]. The combination of erlotinib and the HDACi scriptaid appears to increase H3 K9 acetylation, inhibit the proliferation of erlotinib-resistant GBM cells, and partially restore drug sensitivity. Notably, this regimen also affected additional GBM cell lines regardless of EGFR mutation status [272]. Co-administration of vorinostat and the MEK inhibitor trametinib has demonstrated the ability to suppress malignant features of GBM cells while downregulating BCL-2 family genes and replacing trametinib with the BCL-2 inhibitor navitoclax seems to further enhance apoptosis [273]. Phospholipase D1 (PLD1), a transcriptional target of HDACis, has been implicated in GBM resistance to these agents. Vorinostat can upregulate PLD1 expression through the PKCζ-Sp1 signaling axis, and co-treatment with the PLD1 inhibitor VU0155069 was reported to reverse resistance and further inhibit GBM cell invasion, angiogenesis, and self-renewal [274]. Sorafenib in combination with sodium valproate or vorinostat can trigger GBM cell death via extrinsic apoptotic pathways, although in vivo validation remains limited [275]. The DNA damage response pathway has also been associated with GBM progression. Combined use of vorinostat and the PARP inhibitor olaparib can suppress GBM cell survival, induce cell cycle arrest, and promote apoptosis [276]. Additionally, the Hh signaling pathway appears to interact with HDAC6, and simultaneous inhibition using cyclobenzaprine and Tubastatin A was found to be more effective in reducing GBM cell viability than single agents. This effect may be linked with induced lysosomal stress and disruption of lysosome-autophagosome fusion, thereby potentially impeding GBM progression [277].

Beyond HDACis, combining EZH2 inhibitors with targeted therapies is emerging as a promising strategy. The tazemetostat and PI3 K inhibitor PI-103 combination markedly suppressed malignant phenotypes of GBM cells in vitro [278]. Recent evidence further suggests that EZH2 may regulate pyroptosis in GBM through STAT3 signaling, and treatment with DZNep plus the STAT3 inhibitor SH-4–54 led to increased pyroptosis markers and higher levels of IL-1β and IL-18, indicating that modulating pyroptosis could offer novel therapeutic opportunities [279]. Additionally, the NF-κB inhibitor CAPE in combination with the EZH2 inhibitor EPZ-6438 acted synergistically to suppress GBM progression in both in vitro and subcutaneous mouse models [172]. MYC remains a key oncogene in GBM, with aurora kinase A expression found to correlate with MYC levels in MYC-positive cells. Data indicates that pairing the BET inhibitor JQ1 with the aurora kinase inhibitor MLN8237 results in synergistic antitumor activity [280]. Birabresib (another BET inhibitor), when combined with the PARP inhibitor rucaparib, effectively suppressed GBM malignancy in vitro and in vivo, including in zebrafish and nude mouse models. Notably, administering PARP inhibitors before BET inhibitors appeared to reduce toxicity without compromising efficacy [281]. PRMT5-mediated methylation of hnRNP A1 promotes IRES-dependent translation and contributes to mTOR inhibitor resistance. The PRMT5 inhibitor EPZ015666 has been reported to have synergistic anti-GBM effects in vitro and, when combined with the mTOR inhibitor PP242, in xenograft mouse models [282].

### Histone-modifying agents in immunotherapy combinations

Lysoviruses have emerged as a potential immunotherapeutic strategy for GBM treatment. Pre-treatment of GBM cell lines with VPA has been shown to enhance oncolysis mediated by equine herpesvirus type 1 [283]. In a GBM transplant mouse model, VPA administration appeared to suppress STAT5/T-BET signaling and interferon-γ production, which may inhibit immune cell recruitment and inflammatory responses in natural killer cells, potentially enhancing the efficacy of oncolytic herpes simplex virus [284]. Tubacin was found to enhance the replication and intercellular spread of the oncolytic herpes simplex virus in GBM models [285]. Another study indicated that combining scriptaid and panobinostat with the oncolytic adenovirus Delta24-RGD led to upregulation of αvβ3 integrin, enhanced viral infection, activation of multiple cell death pathways, and amplified antitumor responses in GBM models [286].

EGFR-targeted chimeric antigen receptor T (CAR-T) cell therapy often encounters resistance in GBM. JQ1 has been reported to improve the efficacy of EGFR CAR-T cell therapy by inhibiting BRD4 and reducing the activation of immunosuppressive genes. This combination was associated with enhanced suppression of GBM cell growth and invasion, as well as prolonged survival in mouse models [287]. However, convincing evidence supporting the combination of immune checkpoint inhibitors and histone-modifying agents in GBM remains limited, despite their success in other cancer types. This limitation may be partly attributed to the distinct “cold” immune microenvironment seen in GBM.

### Other targeting combination therapies

Combination therapies targeting histone modifications appear to yield synergistic anti-tumor effects in GBM, particularly when both HDAC and KDM1 A inhibitors are utilized. HDAC inhibition in GBM cells has been reported to result in histone methylation accumulation, which may be reversible by KDM1 A activity. Several studies indicate that combining the KDM1 A inhibitor anti-phencyclidine with HDACis such as vorinostat or abexinostat may synergistically promote cell death in GBM cells [288]. Notably, co-administration of vorinostat and anti-phencyclidine was associated with reduced viability of patient-derived GSCs and showed therapeutic benefit in U87 xenograft models [289]. KDM1 A is also thought to collaborate with the lncRNA HOTAIR in demethylating H3 K4, a process implicated in tumorigenesis. In line with this, the combination of the HOTAIR-EZH2 disruptor AC1Q3QWB with the KDM1 A inhibitor GSK-LSD1 has been shown to induce apoptosis and enhance antitumor responses in xenograft models [290].

Besides KDM1 A-based combinations, pairing BET inhibitors with other histone-modifying drugs may offer additional therapeutic value. For instance, studies suggest that combining BET inhibitors such as JQ1 or OTX015 with panobinostat can markedly inhibit GBM cell proliferation and induce apoptosis, likely by suppressing oncogenic signaling and promoting tumor suppressor gene expression, while also impacting cell metabolism [291, 292]. However, this dual blockade often leads to compensatory upregulation of anti-apoptotic Mcl-1; adding sorafenib to yield a triple combination was found to further suppress tumor growth in patient-derived xenograft models [292]. It is worth noting that panobinostat-OTX015 combinations did not show superior efficacy to OTX015 monotherapy in orthotopic GSCs-derived xenografts, possibly due to limited blood–brain barrier penetration, although better responses were observed in subcutaneous models [293]. Additional mechanisms involve BRD4-mediated STAT3 signaling, where HDAC3 inhibition enhances H3 K27 acetylation and BRD4 recruitment to the GLI1 promoter, boosting transcription. Combined use of the HDAC3 inhibitor RGFP966 and the BET inhibitor JQ1 was found to significantly suppress GSC proliferation, drive apoptosis, inhibit tumor growth in vivo, and synergistically disrupt the Gli1/IL6/STAT3 axis [77, 294].

Further promising results have been seen when combining HDACis with proteasome or metabolic inhibitors. Synergistic induction of apoptosis, increased ROS production, and DNA damage can be achieved with combinations of HDACis (panobinostat, vorinostat, VPA, sodium butyrate) and the proteasome inhibitor bortezomib [295, 296]. Similar effects result from using dacinostat (LAQ824) or TSA alongside MG132 in GBM cells [295]. Combined treatment of panobinostat with the mitochondrial chaperone inhibitor gamitrinib has been reported to suppress tumor metabolism and enhance GBM cell death more effectively than monotherapy [297]. These combinations suggest that HDACis can induce glycolytic reprogramming in GBM cells; subsequent glycolytic inhibition compels reliance on fatty acid oxidation (FAO), and dual therapy with romidepsin (HDAC1/2 inhibitor) and etomoxir (FAO inhibitor) has shown impressive tumor suppression in mouse models [298]. Additionally, BET inhibitors like JQ1, together with the creatine analog CCR, which impairs phosphocreatine synthesis, more effectively halt tumor growth and prolong survival in GBM models, with minimal observed toxicity [299].

Finally, combining histone-modifying agents with cell cycle regulators may offer further benefits. For instance, the combination of the EZH2 inhibitor GSK126 and the CDK4/6 inhibitor abemaciclib disrupts endoplasmic reticulum-mitochondrial homeostasis and upregulates differentiation-associated genes, reinforcing antitumor activity in subcutaneous xenograft models [300]. Similarly, co-treatment with the HOTAIR-EZH2 inhibitor AC1Q3QWB and the CDK4/6 inhibitor palbociclib has been shown to modulate cell cycle and Wnt/β-catenin signaling, curbing GBM cell progression and migration [301]. Moreover, combining the CDK4/6 inhibitor palbociclib with a p300 inhibitor greatly reduced tumor growth in GBM xenografts [37].

## Clinical trials targeting histone modification modifiers in GBM

Clinical application of histone modification modulators in GBM remains at an early stage. Current studies provide valuable insights and a foundation for future clinical trials and drug development (Table 4).

**Table 4:** Small-molecule drugs targeting histone modifiers in clinical trials for GBM

Target	Drug	Treatment Protocol	Patient Group	Phase	Primary Results	Published year	Reference
HDAC	Vorinostat	Vorinostat	Recurrent GBM	II	The mOS was 5.7 months	2009	[302]
HDAC	Romidepsin	Romidepsin	Recurrent GBM	II	The mOS was 8.5 months	2011	[309]
HDAC	Vorinostat	Vorinostat + Bortezomib	Recurrent GBM	II	The mOS was 3.2 months	2012	[305]
HDAC	Panobinostat	Panobinostat + Bevacizumab	Recurrent GBM	II	The mPFS was 5 months, and the mOS was 9 months	2015	[307]
HDAC	Panobinostat	Panobinostat + FSRT	Recurrent HGG (including GBM)	I	Terminated due to poor trial design	2016	[308]
HDAC	Vorinostat	Vorinostat + TMZ + RT	Newly diagnosed GBM	I/II	The OS rate at 15 months was 55.1% with mOS of 16.1 months	2018	[306]
HDAC	Vorinostat	Vorinostat + Bevacizumab	Recurrent GBM	II	6-month PFS was 30.0%, and the mOS was 10.4 months	2018	[303]
HDAC	Valproic Acid	Valproic Acid + TMZ + RT	Newly diagnosed GBM	II	The mPFS was 10.5 months, and the mOS was 29.6 months	2019	[311]
HDAC	Vorinostat	Vorinostat + Erlotinib + TMZ	Recurrent GBM	I/II	Terminated due to unexpected toxicity	2020	[272]
HDAC	Belinostat	Belinostat + RT + TMZ	Newly diagnosed GBM	N/A	The mOS for the belinostat cohort was 18.5 months, while for the control group was 15.8 months (*p* = 0.53)	2022	[314]
BET	OTX015	OTX015	Recurrent GBM	IIa	The dose exploration study for GBM patients was prematurely terminated. due to a lack of efficacy	2016	[315]
BET	Trotabresib	Trotabresib + TMZ + RT	Newly diagnosed GBM	Ib	The RP2D of trotabresib was selected as 30 mg, administered for 4 days followed by a 24-day drug-free period, with good tolerability and safety	2023	[317]
PRMT5	PRT811	PRT811	HGG that have exhausted available treatment options	I	Only one case of grade 3 thrombocytopenia was reported. A durable complete response was observed in a GBM patient with an IDH1 mutation	2021	[318]

*Abbreviations*:*HDAC* histone deacetylase, *GBM* glioblastoma, *HGG* high-grade glioma, *mOS* median overall survival, *mPFS* median progression-free survival, *TMZ* temozolomide, *RT* radiotherapy, *FSRT* fractionated stereotactic re-irradiation therapy, *IDH1* isocitrate dehydrogenase 1 Note: Only common abbreviations are explained below. Other gene/protein abbreviations can be found in the abbreviation list

HDACis are the focus of clinical research in GBM, yet none have achieved regulatory approval thus far. Vorinostat was the first HDACi tested clinically for GBM. In a phase II study in recurrent GBM patients, the median OS was 5.7 months, with grade ≥ 3 toxicities including thrombocytopenia (22%), fatigue (17%), and dehydration (6%) [302]. While vorinostat monotherapy displayed some clinical activity and was generally tolerable, the benefit appeared modest. A phase II trial assessing bevacizumab combined with vorinostat reported a median OS of 10.4 months. Grade 4 adverse events were rare, but this combination did not significantly improve OS compared to bevacizumab alone [303]. Similarly, a multicenter phase II investigation confirmed a lack of improvement in progression-free survival (PFS) and OS from adding vorinostat to bevacizumab in recurrent GBM [304]. When vorinostat was paired with bortezomib, the median OS was only 3.2 months, and over 30% of patients experienced grade ≥ 3 toxicities; outcomes were particularly poor in those previously exposed to bevacizumab [305]. Despite encouraging preclinical data for combined EGFR and HDAC inhibition, a phase I/II study of vorinostat, erlotinib, and TMZ in recurrent GBM was terminated due to unexpected toxicities [272]. For newly diagnosed GBM, vorinostat combined with standard therapy yielded a median OS of 16.1 months but fell short of efficacy benchmarks; notable grade 3/4 toxicities included lymphopenia (32.7%), thrombocytopenia (28.0%), and neutropenia (21.5%) [306]. In another trial, panobinostat with bevacizumab in recurrent high-grade glioma, including GBM, resulted in a median OS of 9 months, with survival outcomes similar to historical controls [307]. A phase I study combining panobinostat with fractionated stereotactic re-irradiation therapy reported a median OS of 16.1 months, though study limitations preclude firm conclusions [308]. Romidepsin produced a median OS of 8.5 months in phase I/II trials for recurrent GBM, but no significant efficacy as a single agent was observed, despite good tolerability [309]. Analyses of VPA in GBM have produced conflicting results. Retrospective studies have associated VPA use during radiotherapy with improved OS [310]. One phase II trial combining VPA with chemoradiotherapy in newly diagnosed GBM reported a median OS of 29.6 months, with manageable hematologic, neurotoxic, and metabolic toxicities [311]. However, pooled analyses of multiple trials did not find significant PFS or OS benefits for radiotherapy plus VPA over historical controls in newly diagnosed patients [312]. Present evidence does not support routine VPA use in these patients absent a seizure indication. Belinostat (PXD-101), a pan-HDACi derived from vorinostat, shows favorable blood–brain barrier penetration in preclinical studies [313]. In a clinical trial, adding belinostat to TMZ and radiotherapy produced a median OS of 18.5 months, versus 15.8 months for controls (*p* = 0.53), showing no statistically significant difference. The regimen was well tolerated. A higher rate of tumor recurrence within the radiation field was noted in belinostat recipients, raising the possibility of a radiosensitizing effect, though this requires further investigation [314].

BET inhibitors have likewise demonstrated preclinical antitumor activity. OTX015, for instance, was effective in xenograft models and able to penetrate brain tumor tissue [133]. Nevertheless, human phase I trials were halted due to insufficient efficacy, likely related to limited CNS drug penetration [315]. The phase I CC-90010-GBM-001 trial showed that trotabresib (CC-90010), a BET inhibitor, reached the CNS in patients with recurrent high-grade gliomas [316]. In the subsequent phase Ib CC-90010-GBM-002 trial, a recommended dosing regimen was established, with the combination considered safe and tolerable alongside standard therapy. While survival outcomes are not yet mature, preliminary clinical activity has been observed [317].

Finally, PRT811, a selective PRMT5 inhibitor with blood–brain barrier permeability, demonstrated preliminary safety and potential efficacy in phase I tested for high-grade glioma, including a durable response in one patient with an IDH1-mutant tumor [318].

## Discussion

Treatment options for GBM remain limited, as conventional modalities—such as surgery, radiotherapy, and chemotherapy—often do not achieve durable disease control, resulting in poor prognoses for many patients [1]. Consequently, there is a pressing need for innovative therapeutic strategies to improve outcomes. Histone modifications, as important epigenetic regulators, have been implicated in GBM progression, therapeutic resistance, modulation of the immune microenvironment, and metabolic reprogramming [28, 43, 85, 123, 212, 247, 298]. This review outlines recent advances in acetylation, methylation, and emerging modifications including lactylation, crotonylation, and succinylation, and provides a relatively comprehensive summary of pharmacological agents targeting various histone modifications, highlighting their possible roles in tumor progression, therapy resistance, signaling crosstalk, and combination treatments [34, 235, 236, 244]. Novel strategies, such as PROTAC-mediated degradation, are also discussed, offering new perspectives for GBM therapy [138].

However, there are still significant limitations in current research and therapeutic approaches related to histone modification. 1) Research on HATs remains relatively limited, especially regarding systematic mechanistic analysis and sufficient clinical evidence; further basic and clinical studies are urgently needed. 2) Although HDACis represent a rapidly developing class of drugs, their broad target profiles result in limited selectivity, frequent off-target toxicity, and the risk of acquired resistance with long-term use. 3) There are yet no standardized protocols for optimizing dosing and scheduling of HDACis inhibitors when combined with radiotherapy, chemotherapy, or immunotherapy, which limits the effectiveness of these combination therapies in clinical practice [272, 287, 313]. 4) The development of targeted agents for HMTs and demethylases is still at an early stage, with limited clinical trial data available. 5) The high heterogeneity and complex cellular composition of GBM make single-target therapies less effective. At the same time, the regulatory network associated with histone modifications is extremely complex, involving multiple enzymes and signaling pathways, further increasing the difficulty of mechanistic studies and drug design [319]. Many existing studies are based on cell lines and animal models, which cannot fully recapitulate the human tumor microenvironment and host interactions—this partly explains the discrepancies between preclinical results and clinical trial outcomes. 7) In addition, the safety, resistance, and potential drug interactions of histone modifier enzyme inhibitors in clinical combination therapies remain unresolved issues.

Recent advances in single-cell sequencing, proteomics, and metabolomics are enhancing our ability to investigate dynamic histone modification changes and their regulatory mechanisms in GBM [320–323]. However, translating these complex multi-omics findings into precise therapeutic strategies presents ongoing challenges. Integrating multi-omics approaches will likely be necessary to better elucidate the role of epigenetic regulation in heterogeneous tumor microenvironments and to identify more reliable therapeutic targets. Future drug development could benefit from prioritizing highly selective, low-toxicity histone-modifying agents, optimizing formulations and dosing schedules, and improving safety and efficacy. Clinical trials should further consider patient genetics, tumor molecular subtypes, and rational drug combinations to advance personalized therapies. Attention to long-term safety, ethical concerns, and real-world follow-up will be important in future investigations.

Although providing a comprehensive overview, our review has several limitations. First, most evidence discussed is derived from preclinical studies, and results from cell and animal models may not fully translate to clinical efficacy or safety in patients. Second, the review primarily focuses on histone acetylation and methylation, with less emphasis on other emerging modifications due to limited available data. Third, included studies vary substantially in their experimental designs, which may limit the comparability and generalizability of findings. Furthermore, the rapidly evolving field may have led to the omission of the most recent developments. Finally, as a narrative rather than a systematic review, quantitative synthesis, and formal evidence grading were not performed. These limitations should be considered when interpreting our findings.

## Conclusion and future perspectives

In summary, histone modifications are key epigenetic regulators in GBM, representing promising therapeutic targets. Despite demonstrating antitumor potential preclinically, clinical applications face challenges due to GBM heterogeneity, limited drug selectivity, and acquired resistance. Future research should clarify histone-modifying enzymes’ roles in GBM tumorigenesis and resistance, develop selective and safe inhibitors, and optimize dosing strategies. Combining traditional therapies with emerging modalities, including immunotherapy, may enhance treatment efficacy. Additionally, developing robust biomarkers for precise patient stratification is vital for personalized therapy advancement. With continuing technological progress and multidisciplinary collaboration, therapies targeting histone modifications may eventually become an important part of strategies to improve outcomes for patients with GBM.

## Data Availability

No datasets were generated or analyzed during the current study.

## Abbreviations

ADRM1: Adhesion regulating molecule 1
APC: Adenomatous polyposis coli
ASIR: Age-standardized incidence rate
ATF4: Activated transcription factor 4
BET: Bromodomain and Extra-Terminal domain
BRD: Bromodomain
BTSC: Brain tumor stem cell
CARM1: Coactivator-Associated Arginine Methyltransferase 1
CAR-T: Chimeric antigen receptor T
CBP: CREB-binding protein
CBTRUS: Central Brain Tumor Registry of the United States
CBX3: Chromobox 3
CCNB1: Cyclin B1
CDC20: Cell division cycle 20
CDK9: Cyclin-dependent kinase 9
CDKN1B: Cyclin-dependent kinase inhibitor 1B
CHK1: Checkpoint kinase 1
CK2: Casein kinase 2
c-Myc: Myelocytomatosis oncogene
CNS: Central nervous system
CREB: Cyclic-AMP response element-binding protein
CYP17 A1: Cytochrome P450 17 A1
DKK1: Dickkopf-1
DZNep: 3-deazaneplanocin A
E2F2: ELL-associated factor 2
E2F7: E2F transcription factor 7
EGFR: Epidermal growth factor receptor
EHMT2, G9a: Euchromatic histone lysine methyltransferase 2
EMT: Epithelial-mesenchymal transition
EZH2: Enhancer of zeste homolog 2
FAO: Fatty acid oxidation
FASN: Fatty acid synthase
FBXW7: F-box and WD repeat domain-containing 7
FDA: US Food and Drug Administration
FKHRL1: Forkhead in rhabdomyosarcoma-like 1
FOXM1: Forkhead box M1
FSRT: Fractionated stereotactic re-irradiation therapy
G6PD: Glucose-6-phosphate dehydrogenase
GBM: Glioblastoma
GDF15: Growth differentiation factor 15
GDNF: Glial cell line-derived neurotrophic factor
Gli1: Glioma-associated oncogene homolog 1
GLP: G9a-like protein
GLUT3: Glucose transporter 3
GNAT: Gcn5-related N-acetyltransferase
GSCs: Glioblastoma stem cells
GSK3β: Glycogen synthase kinase 3β
GTPSCS: Guanosine triphosphate (GTP)-specific succinyl-CoA synthetase
HATs: Histone acetyltransferases
HDACis: Histone deacetylase inhibitors
HDACs: Histone deacetylases
HDMs: Histone demethylases
Hh: Hedgehog
HIF-1α: Hypoxia-inducible factor 1α
HIF-2α: Hypoxia-Inducible factor 2 α
HMTs: Histone methyltransferases
HP1BP3: Heterochromatin protein 1 binding protein 3
Hsp90: Heat shock protein 90
HSPB1: Heat shock protein B1
HTRA1: High-temperature requirement A serine peptidase 1
IARC: International Agency for Research on Cancer
IL-6: Interleukin-6
ISR: Integrated stress response
JAG1: Jagged 1
JAK2: Janus kinase 2
JNK: c-Jun N-terminal kinase
KMTs: Lysine methyltransferases
LDH: Lactate dehydrogenase
lnc TALC: Long non-coding temozolomide-associated lncRNA in glioblastoma recurrence
LUC7L2: LUC7 like 2
MAPK: Mitogen-activated protein kinase
me1: Monomethylation
me2: Dimethylation
me3: Trimethylation
MEK: Mitogen-activated protein kinase kinase
ERK: Extracellular signal-regulated kinase
MELK: Maternal embryonic leucine zipper kinase
MGMT: O⁶-methylguanine-DNA methyltransferase
miR-3189: microRNA-3189
MKK7: Mitogen-activated protein kinase kinase 7
MLH1: MutL homolog 1
MLL1: Mixed-lineage leukemia 1
MOZ: Monocytic leukemia zinc finger protein
MRP1: Multidrug resistance protein 1
MST1: Mammalian sterile 20-like kinase 1
MT1-MMP: Matrix metalloproteinase 14
Mtor: Mammalian target of rapamycin
NAD⁺: Nicotinamide adenine dinucleotide
NADPH: Nicotinamide adenine dinucleotide phosphate
NFAT2: Nuclear factor of activated T cells 2
NFYB: Nuclear transcription factor Y subunit β
NF-κB: Nuclear Factor kappa-light-chain-enhancer of activated B cells
NKD1: Naked cuticle homolog 1
Notch1: Neurogenic locus notch homolog protein 1
Oct4: Octamer-binding transcription factor 4
OS: Overall survival
p21: Cyclin-dependent kinase inhibitor 1A
p300: E1A binding protein p300
p53: Tumor protein p53
PCAF: p300/CBP-associated factor
PDK1: 3-phosphoinositide-dependent protein kinase 1
PFS: Progression-free survival
PI3 K: Phosphatidylinositol 3-kinase/protein kinase B
AKT: Phosphatidylinositol 3-kinase/protein kinase B
PLD1: Phospholipase D1
PRC2: Polycomb repressive complex 2
PRMTs: Protein arginine methyltransferases
PROTAC: Proteolysis targeting chimera
PTEN: Phosphatase and tensin homolog
RBBP4: Retinoblastoma-binding protein 4
RBP: CRNA-binding protein
RCC1: Regulator of chromosome condensation 1
REST: Repressor element-1 silencing transcription factor
ROS: Reactive oxygen species
SATB2: Special AT-rich sequence-binding protein 2
SCNN1 A: Sodium channel epithelial 1α
SDC1: Syndecan-1
SETDB1: SET domain bifurcated histone lysine methyltransferase 1
SHH: Sonic hedgehog
SHMT2: Serine hydroxymethyltransferase 2
SKI: Ski proto-oncogene
SLC30A3: Solute carrier family 30 member 3
SLC7A11: Solute carrier family 7 member 11
SMAD1: SMAD family member 1
SOX2: Sex determining region Y-box 2
Sp1: Specificity protein 1
ST7: Suppressor of tumorigenicity 7
STAT3: Signal transducer and activator of transcription 3
STUB1: STIP1 homology and U-box containing protein 1
SUV39H1: Suppressor of variegation 3-9 homolog 1
TAZ: Transcriptional co-activator with PDZ-binding motif
TFPI2: Tissue factor pathway inhibitor 2
TGF-β: Transforming growth factor β
Tip60: Tat-interacting protein 60
TMZ: Temozolomide
TRAF4: TNF receptor-associated factor 4
TRAF6: TNF receptor-associated factor 6
TRAIL: TNF-related apoptosis-inducing ligand
TRAP1: Tumor necrosis factor receptor-associated protein 1
TRIM24: Tripartite motif containing 24
TSA: Trichostatin A
UPR: Unfolded protein response
USP22: Ubiquitin-specific protease 22
VEGF: Vascular endothelial growth factor
VPA: Valproic acid
WDR5: WD repeat domain 5
WHO: World Health Organization
Wnt: Wingless-related integration site
WNT7B: WNT family member 7B
YTHDF: YTH N6-methyladenosine RNA binding protein 2
ZEB1: Zinc finger E-box binding homeobox 1
α-KGDH: α-ketoglutarate dehydrogenase
